# Supplementary material for: Adverse Effects of Angiotensin-Converting Enzyme Inhibitors in Humans: A Systematic Review and Meta-Analysis of 378 Randomized Controlled Trials
Source: Int J Environ Res Public Health. 2022 Jul 8;19(14):8373. doi: 10.3390/ijerph19148373 (PMC9324875; doi:10.3390/ijerph19148373)
Supplement: Supplementary file 1 [file ijerph-19-08373-s001.zip › Figure S1.pdf]

Figure S1 Risk-of-bias assessments of the included studies

Study (Year)	D1	D2	D3	D4	D5	Overall
Agostoni (1993)	−	−	+	+	−	−
Ahimastos (2007)	+	+	+	+	+	+
Ahimastos (2013)	+	+	+	+	+	+
Ahimastos (2014)	+	+	+	+	+	+
Ajayi (1989)	−	+	+	+	+	−
Allemann (1992)	+	−	+	+	+	−
Ambrosioni (1995)	+	+	+	+	+	+
Anderson (1991)	+	−	+	+	+	−
Anderson (1994)	−	−	+	+	+	−
Annane (1996)	+	+	+	+	+	+
Arashi (2020)	+	+	+	+	+	+
Avtar (1999)	−	+	+	+	+	−
Babu–Narayan (2012)	+	+	+	+	+	+
Baker (1988)	−	+	+	+	+	−
Barr (1997)	+	+	+	+	+	+
Beckett (2008)	+	+	+	+	+	+
Beckingham (1995)	+	−	+	+	+	−
Bedogna (1990)	−	+	+	+	+	−
Beller (1995)	+	+	+	+	+	+
Bellissant (1994)	+	−	+	+	+	−
Benedict (1995)	+	−	+	+	+	−
Berg (1995)	+	−	−	−	+	−
Bergstrand (1985)	−	−	+	+	+	−

Study (Year)	D1	D2	D3	D4	D5	Overall
Bertrand (2015)	+	+	+	+	+	+
Bicknell (2016)	+	+	+	+	+	+
Black (1997)	+	+	+	+	+	+
Böhlen (1996)	+	−	+	+	+	−
Bohm (2005)	+	+	+	+	+	+
Bohm (2010)	+	+	+	+	+	+
Bojestig (2001)	+	+	+	+	+	+
Bonnemeier (2007a)	−	−	+	−	−	X
Bonnemeier (2007b)	+	X	X	+	+	X
Borghi (1993)	−	+	+	+	+	−
Borghi (1997)	+	+	+	+	+	+
Borghi (1998)	+	+	+	+	+	+
Borghi (2007)	+	+	+	+	+	+
Bosh (2006)	+	+	+	+	+	+
Brown (1990)	+	+	+	+	+	+
Brown (1995)	+	+	−	+	+	−
Brugts (2014)	+	+	+	X	+	X
Bruijn (1994)	−	+	+	+	+	−
Bull (2015)	+	+	+	+	+	+
Burggraaf (1998)	+	+	+	+	+	+
Burris (1991)	+	−	+	+	+	−
Bussmann (1987)	+	+	+	+	+	+
Caletti (2011)	+	+	+	+	+	+

Domains:
D1: Bias due to randomisation.
D2: Bias due to deviations from intended intervention.
D3: Bias due to missing data.
D4: Bias due to outcome measurement.
D5: Bias due to selection of reported result.

Judgement
X High
- Some concerns
+ Low

Study (Year)	D1	D2	D3	D4	D5	Overall
Campbell (1985)	-	+	+	+	+	-
Capek (1994)	+	+	+	-	-	-
CAPTOPRIL MULTICENTER RESEARCH GROUP (1983)	+	+	+	+	+	+
Cardillo (1994)	+	-	+	+	+	+
Carlsen (1989)	X	-	+	+	+	X
Carp (1994)	-	+	+	+	+	-
Carré (1992)	+	+	+	+	+	+
Challenor (1991)	-	+	+	+	+	-
Chalmers (1987)	+	+	+	+	+	+
Chatellier (1987)	-	+	+	+	+	-
Chen (2002)	+	+	+	+	+	+
Cheung (1999)	+	-	+	+	+	-
Cheung and Lau (1999)	+	+	+	+	+	+
Chinese Cardiac Study (CCS–1) (1995)	-	X	+	+	-	X
Chrysant (1985)	-	+	+	+	+	-
Chrysant (1993)	+	+	+	+	+	+
Chrysant (2000)	+	-	-	-	+	-
Chrysant (1996)	+	+	+	+	+	+
Chrysant (1997)	+	+	+	+	+	+
Circo (1995)	+	-	-	+	+	-
Cleland (1985)	+	-	+	+	-	-
Cleland (1991)	+	+	+	+	+	+
Cleland (1999)	+	+	+	+	+	+

Study (Year)	D1	D2	D3	D4	D5	Overall
Cockerham (2017)	+	-	+	+	+	-
Colfer (1992)	+	+	-	-	+	-
CONSENSUS Trial Study Group (1987)	+	+	+	+	+	+
Coppo (2007)	+	+	+	+	+	+
Corbalán (1990)	-	-	+	+	-	-
Cordonnier (1999)	+	+	+	+	+	+
Costagliola (1995)	+	+	+	+	+	+
Crepaldi (1998)	+	+	+	+	+	+
Dahlof (2005)	+	+	+	+	+	+
Dalsgaard (2014)	+	+	+	+	+	+
Darasz (1995)	+	+	+	+	+	+
DeQuattro (1997)	+	+	+	+	+	+
DeQuattro and Lee (1997)	+	+	+	+	+	+
Di Marco (2010)	+	+	+	+	+	+
Dickstein (1991a)	+	+	+	+	+	+
Dickstein (1991b)	-	-	-	+	-	X
Dicpinigaitis and Dobkin (1996)	+	+	+	+	+	+
Dössegger (1993)	+	-	+	+	+	-
Drayer and Weber (1983)	-	-	-	+	+	-
Duboc (2005)	+	+	+	+	+	+
Duboc (2007a)	+	+	+	+	+	+
Duboc (2007b)	+	+	+	+	+	+
Dyker (1997)	+	+	+	+	+	+

Domains:
D1: Bias due to randomisation.
D2: Bias due to deviations from intended intervention.
D3: Bias due to missing data.
D4: Bias due to outcome measurement.
D5: Bias due to selection of reported result.

Judgement
X High
\- Some concerns
\+ Low

Study (Year)	D1	D2	D3	D4	D5	Overall
Ekholm (2002)	+	−	+	+	+	−
Erhardt (1995)	+	+	+	+	+	+
Evans (1987)	+	+	+	+	+	+
Eveson (2007)	+	+	+	+	+	+
Fagard (2001)	+	+	+	+	+	+
Faxon (1995)	+	+	+	+	+	+
Ferrari (2006)	+	+	+	+	+	+
Flather (1994)	+	+	+	+	+	+
Ford (1993)	−	+	+	+	+	−
Forette (1987)	−	+	+	+	+	−
Foucan (1998)	+	+	+	+	+	+
Fox (2003)	+	+	+	+	+	+
Franciosa (1985)	−	−	+	+	−	−
Frcka and Lader (1988)	+	+	+	+	+	+
French (1999)	−	+	X	+	−	X
Fuller (1987)	−	−	X	+	+	X
Galcera–Tomas (1993)	−	−	+	+	+	−
Gall (1992)	+	+	+	+	+	+
Gansevoort (1994)	−	−	+	+	+	−
Gerritsen (1998)	+	+	−	+	+	−
Gianni (2007)	+	+	+	+	+	+
Gilbert (1993)	+	−	+	+	+	−
Gleerup (1996)	+	−	+	+	+	−

Study (Year)	D1	D2	D3	D4	D5	Overall
Gliddon (2007)	+	+	+	+	+	+
Gomez (1989)	+	+	+	+	+	+
González–Fernández (1993)	−	−	+	+	−	−
Gordon (1997)	+	+	+	+	+	+
Gøtzsche (1992)	+	−	+	+	+	−
Gradman (1995)	+	+	+	+	+	+
Gross (2020)	+	+	+	+	+	+
Guazzi (1999)	+	+	+	+	+	+
Guglin (2019)	+	+	+	+	+	+
Guillevin (1981)	−	−	+	−	−	X
Güntzel (1991)	−	+	+	+	+	−
Gupta (1990)	+	+	+	+	+	+
Hampton (1998)	+	+	+	+	+	+
Hatazawa (2004)	−	−	+	+	+	−
Heart Outcomes Prevention Evaluation (HOPE) Study Investigators (2000)	+	+	+	+	+	+
Henderson (1994)	−	+	+	+	+	−
Hernández (2000)	−	+	+	+	+	−
Herrlin (1990)	−	+	+	+	+	−
Heuvel (1997)	+	+	+	+	+	+
Holdaway (1994)	+	−	+	+	+	−
Holwerda (1996)	+	+	+	+	+	+
Hommel (1986)	+	+	+	+	+	+
Homuth (1993)	+	+	+	+	+	+

Domains:
D1: Bias due to randomisation.
D2: Bias due to deviations from intended intervention.
D3: Bias due to missing data.
D4: Bias due to outcome measurement.
D5: Bias due to selection of reported result.

Judgement
X High
- Some concerns
+ Low

Study (Year)	D1	D2	D3	D4	D5	Overall
Hong (2018)	+	+	+	+	+	+
Hou (2006)	+	+	+	+	+	+
Hoy (2019)	+	+	+	+	+	+
Hryniewicz (2005)	+	−	+	+	+	−
Hsu (2009)	+	+	+	+	+	+
Hsu (2010)	+	+	+	+	+	+
Hutcheon (2002)	+	+	+	+	+	+
ISIS–4 (Fourth International Study of Infarct Surviv Collaborative Group (1995)	+	+	+	+	+	+
Janbabai (2017)	+	+	+	+	+	+
Janini (1988)	+	+	+	+	+	+
Javannnard (2011)	+	+	+	+	+	+
Jerums (2001)	+	+	+	+	+	+
Jirasirirojanakorn (1998)	−	+	+	+	+	−
Jones–Burton (2010)	+	+	+	+	+	+
Jose (2006)	+	+	+	+	+	+
Joy (1987)	+	+	+	+	+	+
Kaski (1994)	+	+	+	+	+	+
Katayama (2002)	+	+	+	+	+	+
Katz (1991)	X	+	+	+	+	X
Kaufman (1993)	−	−	+	+	−	−
Kim (2015)	+	−	+	+	+	−
Kitzman (2010)	+	+	+	+	+	+
Kjekshus and Swedberg (1988	+	+	+	+	+	+

Study (Year)	D1	D2	D3	D4	D5	Overall
Kjekshus and Swedberg (1989)	+	+	+	+	+	+
Klapholz (2001)	+	−	+	−	−	−
Kleber (1992)	+	+	+	+	+	+
Kloke (1990)	−	−	+	+	−	−
Kondowe (1987)	+	+	+	+	+	+
Konstam (1992)	−	−	−	−	+	X
Konstam (1993)	+	+	−	+	+	−
Kontopoulos (1999)	−	+	+	−	−	−
Koskinen (1988)	+	−	+	+	+	−
Kouatli (1997)	+	+	+	+	+	+
Krysiak and Okopien (2008)	+	+	+	+	+	+
Krysiak and Okopien (2011)	+	−	−	+	+	−
Küppers (1997)	+	+	+	+	+	+
Kuschnir (1996)	−	+	+	+	+	−
Kvetny (2001)	+	−	+	+	+	−
Labinjoh (2001)	+	+	+	+	+	+
Lang (1995)	+	+	+	+	−	−
Laurent (2015)	+	+	+	+	+	+
Leary (1985)	−	−	+	+	+	−
Lechat (1993)	−	+	+	+	+	−
Lee (1999)	+	+	+	+	+	+
Lees and Reid (1987)	−	+	+	+	+	−
Leonetti (1991)	−	+	+	+	+	−

Domains:
D1: Bias due to randomisation.
D2: Bias due to deviations from intended intervention.
D3: Bias due to missing data.
D4: Bias due to outcome measurement.
D5: Bias due to selection of reported result.

Judgement
X High
- Some concerns
+ Low

Study (Year)	D1	D2	D3	D4	D5	Overall
Lerch (1999)	-	-	+	+	+	-
Levine and Applegate (1997)	+	+	+	+	+	+
Lewis (1993)	+	+	+	+	+	+
Lewis (1996)	+	+	+	+	+	+
Lewis (1988)	+	+	+	+	+	+
Liao (2003)	+	-	+	+	+	-
Lonn (2001)	+	+	+	+	+	+
Lonn (2002)	X	-	+	+	-	X
Lonn (2009)	+	+	+	+	+	+
Lough (1998)	+	X	X	+	+	X
Luca (1993)	+	+	+	+	+	+
Luksch (2006)	+	+	+	+	+	+
MacDonald (1990)	+	+	+	+	+	+
MacFadyen (1997)	+	+	+	+	+	+
MacFadyen and Struthers (1997)	+	+	+	+	+	+
Maclean (1989)	+	+	+	+	+	+
MacMahon (2000)	+	+	+	+	+	+
Malik (1998)	+	+	+	+	+	+
Mancia (1992)	+	+	+	+	+	+
Mancia (2014)	+	+	+	+	+	+
Mandelbrot (2015)	+	-	+	+	+	-
Marketou (2008)	+	-	+	-	+	-
Marney (2010)	+	-	+	+	+	-

Study (Year)	D1	D2	D3	D4	D5	Overall
Marre (2004)	+	+	-	+	+	-
Martinuzzi (2008)	+	+	+	+	+	+
Maschio (1996)	+	+	+	+	+	+
Mathur (2020)	+	+	+	+	+	+
Matsumoto (2006)	-	-	X	+	+	X
Mauer (2009)	+	+	+	+	+	+
Mazayev (1998)	+	+	+	+	+	+
Mbah (2012)	+	+	+	+	+	+
McNabb (1986)	-	-	+	+	-	-
Mersey (1993)	+	-	-	+	+	-
Messerli (1998)	+	+	+	+	+	+
Metelitsa (1992)	-	+	+	+	+	-
Miller (1994)	+	-	+	+	+	-
Mitrovic (1996)	+	+	+	+	+	+
Morgan (1995)	+	+	+	-	-	-
Moser (1991)	+	+	+	+	+	+
Moser and Lunn (1982)	-	+	+	+	+	-
Moyses and Higgins (1992)	-	+	+	+	+	-
Muiesan (1993)	+	+	+	+	+	+
Mullen (1998)	+	-	+	+	-	-
Mulligan (1988)	+	+	+	+	+	+
Nabel (1991)	+	+	+	+	+	+
Naeije (1993)	-	+	+	+	+	-

Domains:
D1: Bias due to randomisation.
D2: Bias due to deviations from intended intervention.
D3: Bias due to missing data.
D4: Bias due to outcome measurement.
D5: Bias due to selection of reported result.

Judgement
X High
- Some concerns
+ Low

Study (Year)	D1	D2	D3	D4	D5	Overall
Nagamia (2007)	+	+	+	+	+	+
Nalbantgil (1998)	−	+	+	+	+	−
Naranjo (1991)	+	+	+	+	+	+
Nazir (2005)	+	+	+	+	+	+
Neaton (1993)	+	+	+	+	+	+
Nicholls (1984)	−	+	+	+	+	−
Nicolas (2012)	+	+	+	+	+	+
Northridge (1993)	+	+	+	+	+	+
Northridge (1999)	+	+	+	+	+	+
O'Hare (2000)	+	+	+	+	+	+
O'Neill (1992)	+	+	+	+	−	−
Olajide and Lader (1985)	+	+	+	+	+	+
Oldroyd (1991)	−	+	+	−	−	−
Oosterga (2001)	−	+	−	+	+	−
Oparil (1999)	+	+	+	+	−	−
Ordaz–Medina (2010)	+	+	+	+	+	+
Ormesher (2020)	+	+	+	+	+	+
Oshima (1997)	+	−	−	+	+	−
Parati (2014)	+	+	+	+	+	+
Pasquale (1993)	+	−	+	+	+	−
Paster (1998)	+	+	+	+	+	+
Patat (1989)	+	+	+	+	+	+
Patel (1996)	−	−	+	+	+	−

Study (Year)	D1	D2	D3	D4	D5	Overall
Pauly (2011)	+	+	+	+	+	+
Pepine (2003)	+	+	+	+	+	+
Perez–Vasquez (2020)	+	+	+	+	+	+
Persson and Stimpel (1996)	−	+	+	+	+	−
Petrie (2000)	−	+	+	+	+	−
Pfeffer (1988)	−	+	+	+	−	−
Pfeffer (1992)	+	+	+	+	+	+
Pitt (2001)	X	+	+	+	−	−
Pittrow (1997)	+	+	+	+	+	+
Pituskin (2017)	+	+	+	+	+	+
Pool (1997)	+	+	+	+	+	+
Pool (2001)	+	+	+	+	+	+
Pouleur (1992)	+	+	+	+	+	+
Pouleur (1993)	−	−	+	+	+	−
Prichard (2002)	+	+	+	+	+	+
Prisant (1998)	−	−	+	+	+	−
Prisant (1999)	−	+	+	+	+	−
PROGRESS Collaborative Group (2001)	+	+	+	+	+	+
Puig (2007)	+	+	+	+	−	−
Rahman (2007)	+	−	+	+	+	−
Rahman (2010)	+	−	+	+	+	−
Rake (2001)	+	+	+	+	+	+
Ratnasabapathy (2003)	+	−	+	+	+	−

Domains:
D1: Bias due to randomisation.
D2: Bias due to deviations from intended intervention.
D3: Bias due to missing data.
D4: Bias due to outcome measurement.
D5: Bias due to selection of reported result.

Judgement
X High
- Some concerns
+ Low

Study (Year)	D1	D2	D3	D4	D5	Overall
Ravid (1993)	+	+	+	+	+	+
Ravid (1998)	+	+	+	+	+	+
Ray–Chaudhuri (1993)	−	+	+	+	−	−
Reisin (1997)	+	+	+	+	+	+
Richer (1987)	+	+	+	+	+	+
Riegger (1990)	−	+	+	+	+	−
Riegger (1991)	−	+	−	+	+	−
Rodrigues (2008)	+	+	+	+	+	+
Rodriguez–Granillo (2007)	+	+	+	+	+	+
Rosenbaek (2018)	+	+	+	+	+	+
Rouleau (2008)	+	+	+	+	+	+
Rubin (1984)	−	−	−	−	+	X
Ruggenenti (2004)	+	+	+	+	+	+
Ruggenenti (2011)	+	+	+	+	+	+
Ruilope (2004)	+	+	+	+	+	+
Rustin (1987)	−	+	+	+	+	+
Rutledge (1988)	+	+	+	+	+	+
Saito (1990)	+	+	+	+	+	+
Sampaio (2005)	+	+	+	+	+	+
Sassano (1987)	−	+	+	+	+	−
Saul (2013)	+	+	+	+	+	+
Schill (1994)	+	+	+	+	+	+
Schmidt (1992)	−	+	+	+	+	−

Study (Year)	D1	D2	D3	D4	D5	Overall
Schneider (1999)	−	−	+	+	+	−
Schrader (2001)	+	+	+	+	+	+
Seneviratne (1994)	+	+	+	+	−	−
Shahin (2013)	+	−	+	+	+	−
Sharifipour (2016)	+	−	+	+	+	−
Sharpe (1984)	−	+	+	+	+	−
Shaw (2014)	+	+	+	+	+	+
Sigurdsson (1994)	+	−	−	+	+	−
Silber (2004)	+	+	+	+	+	+
Simon (1989)	−	+	+	+	+	−
Soejima (1996)	−	X	+	+	−	X
Sonbolestan (2013)	+	+	+	+	+	+
Speel (2005)	+	+	+	+	+	+
Squire (1994)	+	+	+	+	+	+
Steinman (1993)	+	+	+	+	+	+
Stephan (1996)	−	−	+	+	+	−
Stimpel (1996)	+	+	+	+	+	+
Strozzi (1987)	−	−	−	−	−	X
Sudoh (1993)	+	+	+	+	+	+
Sullivan (1979)	−	+	+	+	+	−
Sullivan (1988)	−	−	+	+	+	−
Sumukadas (2007)	+	+	+	+	+	+
Sumukadas (2018)	+	+	+	+	+	+

Domains:
D1: Bias due to randomisation.
D2: Bias due to deviations from intended intervention.
D3: Bias due to missing data.
D4: Bias due to outcome measurement.
D5: Bias due to selection of reported result.

Judgement
X High
- Some concerns
+ Low

Study (Year)	D1	D2	D3	D4	D5	Overall
Swedberg (1990)	−	+	X	+	+	X
Taddei (1995)	+	+	+	+	+	+
Taylor (1990)	+	+	+	+	+	+
Teo (2000)	+	+	+	+	+	+
The Acute Infarction Ramipril Efficacy (AIRE) Study Investigators (1993)	+	+	+	+	+	+
The EUCLID study group (1997)	+	+	+	+	+	+
The GISEN Group (Gruppo Italiano di Studi Epidemiologici in Nefrologia) (1997)	+	+	+	+	+	+
The Treatment of Mild Hypertension Research Group (1991)	+	+	+	+	+	+
The Veratran Study Group (1997)	+	+	+	+	+	+
Thind (1985)	+	+	+	+	+	+
Thürig (1995)	+	+	+	+	+	+
Tohmo (1993)	−	+	+	+	+	−
Tong (2006)	+	+	+	+	+	+
Toto (1996)	−	+	+	+	+	−
Trevisan and Tiengo (1995)	+	+	+	+	+	+
Tsang (2006)	+	+	+	+	+	+
Tuininga (2000)	+	+	+	+	+	+
Turner (2006)	−	+	+	+	+	−
Tzourio (2003)	+	+	+	+	+	+
van Kooij (2006)	+	+	+	+	+	+
Veldhuisen (1998)	+	+	+	+	+	+
Verrico (2016)	+	+	+	+	+	+
Veterans Administration Co-Operative Study Group on Antihypertensive Agents (1982)	−	−	+	+	−	−
Veterans Administration Cooperative Study Group on Antihypertensive Agents (1984)	+	−	−	+	+	−
Viberti (1994)	+	+	+	+	+	+
Vlasses (1982)	−	X	+	X	+	X
Vongterapak (1998)	+	−	+	+	+	−
Waeber (1999)	+	+	+	+	+	+
Walter (2002)	+	+	+	+	+	+
Walters (2001)	+	+	+	+	+	+
Walters (2004)	+	+	+	+	+	+
Wang (2012)	+	+	+	+	+	+
Wang (2016)	+	+	+	+	+	+
Webster (1985a)	−	+	+	+	+	−
Webster (1985b)	−	−	+	−	−	X
Weinberger (1983)	+	+	+	X	+	+
Weir and Saunders (1998)	+	+	+	+	+	+
West (1991)	+	−	+	+	+	−
White (1988)	−	−	+	+	+	−
White (1995)	+	+	+	+	+	+
White (2002)	+	+	+	+	+	+
Wiegmann (1992)	+	+	+	+	+	+
Wilkins (1983)	−	+	+	+	+	−
Willenheimer (2001)	+	−	+	+	+	−
Willoughby (2012)	+	+	+	+	+	+
Winer (1990)	+	−	+	+	+	−

Domains:
D1: Bias due to randomisation.
D2: Bias due to deviations from intended intervention.
D3: Bias due to missing data.
D4: Bias due to outcome measurement.
D5: Bias due to selection of reported result.

Judgement
X High
- Some concerns
+ Low

Study (Year)	D1	D2	D3	D4	D5	Overall
Wyld (1994)	+	−	+	+	+	−
Yu (2006)	+	+	+	+	+	+
Yusuf (1991)	+	+	+	+	+	+
Yusuf (1992)	+	+	+	+	+	+
Yusuf (2000)	+	+	+	+	+	+

Study (Year)	D1	D2	D3	D4	D5	Overall
Zamboulis (1996)	−	−	+	+	+	−
Zannad (2006)	+	+	+	+	+	+
Zee (1998)	+	+	+	+	+	+
Zeng (1998)	−	+	+	+	+	−
Zi (2003)	+	+	+	+	+	+

Domains:
D1: Bias due to randomisation.
D2: Bias due to deviations from intended intervention.
D3: Bias due to missing data.
D4: Bias due to outcome measurement.
D5: Bias due to selection of reported result.

Judgement
X High
- Some concerns
+ Low
