# Supplementary material for: Adverse Effects of Angiotensin-Converting Enzyme Inhibitors in Humans: A Systematic Review and Meta-Analysis of 378 Randomized Controlled Trials
Source: Int J Environ Res Public Health. 2022 Jul 8;19(14):8373. doi: 10.3390/ijerph19148373 (PMC9324875; doi:10.3390/ijerph19148373)
Supplement: Supplementary file 1 [file ijerph-19-08373-s001.zip › Table S1.pdf]

Table S1 Characteristics of the included studies

Study	Underlying condition	Gender (M/F)	Age (years)	Ethnics	Study design	Location	ACE inhibitors	Dosage (mg/day)	Treatment duration
Agostoni (1993)	Uncomplicated essential hypertension	14 (8/6)	51.7 ± 9.7	N/A	N/A	Italy	Captopril	50 mg/day	1 day
Ahimastos (2007)	Marfan syndrome	17 (13/4)	33.0 ± 6.0	N/A	Parallel	Australia	Perindopril	8 mg/day	24 weeks
Ahimastos (2013)	Peripheral artery disease	212 (177/35)	65.5 ± 6.2	N/A	Parallel	Australia	Ramipril	10 mg/day	24 weeks
Ahimastos (2014)	Peripheral artery disease with intermittent claudication	165 (137/28)	65.3 ± 6.7	N/A	Parallel	Australia	Ramipril	10 mg/day	24 weeks
Ajayi (1989)	Congestive heart failure	12 (5/7)	49.2 ± 16.1	African	Parallel	Nigeria	Enalapril	10 mg/day	8 days
Allemann (1992)	None	24 (24/0)	25.0 ± 4.1	N/A	Parallel	N/A	Fosinopril	20 mg/day	3 weeks
Ambrosioni (1995)	Acute myocardial infarctions not eligible for thrombolytic therapy	1556 (1128/428)	N/A	N/A	Parallel	Italy	Zofenopril	15-60 mg/day	6 weeks
Anderson (1991)	High diastolic blood pressure	220 (139/81)	47.6 ± 10.9	White and Others	Parallel	United States	Fosinopril	10, 40, and 80 mg/day	4 weeks for short term and 10-13 months for long term
Anderson (1994)	Essential hypertension	20 (20/0)	N/A	N/A	Cross-over	N/A	Enalapril and Peridonril	10, 20 mg/day of enalapril and 4, 8 mg/day of perindopril	1 day
Annane (1996)	Congestive heart failure	20 (11/9)	77.0 ± 8.0	N/A	Parallel	N/A	Enalapril	1 mg IV	2 hours
Arashi (2020)	Heart transplantation	91 (62/29)	52.5 ± 15.9	N/A	Parallel	United States	Ramipril	10-20 mg/day	3 years

Avtar (1999)	None	48 (N/A)	N/A	N/A	Parallel	N/A	Captopril	6.25, 12.5, and 25 mg/day	1 day
Babu-Narayan (2012)	Repaired tetralogy of fallot and pulmonary regurgitation	64 (39/25)	31.1 ± 10.1	N/A	Parallel	N/A	Ramipril	2.5-10 mg/day	6 months
Baker (1988)	Hypertension	16 (10/6)	N/A	N/A	Cross-over	N/A	Captopril	25 mg/day	4 weeks
Barr (1997)	Mild congestive heart failure secondary to coronary artery disease	41 (38/3)	66.0 ± 8.0	N/A	Parallel	N/A	Enalapril	2.5-20 mg/day	1 year
Beckett (2008)	Elderly	3845 (1519/2326)	83.6 ± 3.2	N/A	Parallel	West and Eastern Europe, China, Australasia, and North Africa	Perindopril	2-4 mg/day	1.8 year
Beckingham (1995)	Posttransplant erythrocytosis	25 (N/A)	N/A	N/A	Parallel	N/A	Enalapril	2.5 mg/day	4 months
Bedogna (1990)	Chronic glomerulopathies and preserved renal function	20 (15/5)	35.0 ± 11.8	N/A	Cross-over	Italy	Benazepril	10 mg/day	3 months
Beller (1995)	Heart failure	193 (145/48)	N/A	Black and Non-black	Parallel	N/A	Lisinopril	5-20 mg/day	12 weeks
Bellissant (1994)	None	6 (6/0)	22.7 ± 1.7	N/A	Cross-over	N/A	Lisinopril	5 and 20 mg/day	1 weeks
Benedict (1995)	Left ventricular dysfunction	462 (400/62)	60.3 ± 10.2	Black, White, and Others	Parallel	N/A	Enalapril	N/A	40 months
Berg (1995)	Heart failure and chronic atrial fibrillation	30 (18/12)	68.0 ± 7.2	N/A	Parallel	Netherlands	Lisinopril	2.5-10 mg/day	12 weeks
Bergstrand (1985)	Mild to moderate essential hypertension	91 (54/37)	N/A	Caucasian	Parallel	Sweden	Enalapril	2.5, 5, 10, 20, and 40 mg/day	3 weeks

Bertrand (2015)	Low cardiovascular risk with stable coronary artery disease and without heart failure	7534 (6396/1138)	59.4 ± 9.3	N/A	Parallel	United Kingdom	Perindopril	4-8 mg/day	4 years
Bicknell (2016)	Small abdominal aortic aneurysm	152 (145/7)	71.1 ± 7.2	Caucasian	Parallel	England	Perindopril	10 mg/day	2 years
Black (1997)	Essential hypertension	370 (225/145)	53.9 ± 11.2	White, Black, and Others	Parallel	N/A	Lisinopril	10-20 mg/day	4 weeks
Böhlen (1996)	Overweight with essential hypertension	30 (19/11)	47.9 ± 12.0	N/A	Parallel	Switzerland	Perindopril	4 mg/day	6 weeks
Bohm (2005)	Atherosclerosis	37 (30/7)	66.8 ± 8.5	N/A	Parallel	Sweden	Ramipril	10 mg/day	3 month
Bohm (2010)	Cardiovascular disease	602 (N/A)	N/A	N/A	Parallel	N/A	Ramipril	N/A	54 months
Bojestig (2001)	Type 1 diabetes	55 (41/14)	39.7 ± 9.7	N/A	Parallel	Sweden	Ramipril	1.25 and 5 mg/day	2 years
Bonnemeier (2007a)	Acute myocardial infarction	15 (11/4)	62.3 ± 11.1	N/A	Parallel	N/A	Enalaprilat	50 µg intracoronary	1 day
Bonnemeier (2007b)	Acute myocardial infarction	15 (11/4)	62.4 ± 11.0	N/A	Parallel	N/A	Enalapril	50 µg intracoronary	1 day
Borghi (1993)	Acute myocardial infarction	20 (15/5)	61.1 ± 8.7	N/A	Parallel	Italy	Zofenopril	15-60 mg/day	1 day
Borghi (1997)	Acute myocardial infarction	285 (236/49)	60.1 ± 8.5	N/A	Parallel	Italy	Fosinopril	5-20 mg/day	2 years
Borghi (1998)	Acute myocardial infarction	285 (236/49)	60.1 ± 8.5	N/A	Parallel	Italy	Fosinopril	5-20 mg/day	3 months
Borghi (2007)	Myocardial infarction	349 (289/60)	58.0 ± 9.9	N/A	Parallel	Italy and Europe	Zofenopril	30-50 mg/day	6 months
Bosh (2006)	Impaired fasting glucose levels or impaired glucose tolerance	5,269 (2,149/3,120)	54.7 ± 10.8	N/A	Factorial design	N/A	Ramipril	5-15 mg/day	3 years
Brown (1990)	Hypertension	20 (N/A)	N/A	N/A	Parallel	United Kingdom and France	Perindopril	4 mg/day	4 weeks

Brown (1995)	Mild to moderately severe chronic heart failure	241 (192/49)	62.0 ± 9.5	White, Black, and Others	Parallel	United States	Fosinopril	10-20 mg/day	24 weeks
Brugts (2014)	Vascular disease	23973 (N/A)	N/A	Caucasia and Asian	Parallel	N/A	Perindopril	4-8 mg/day	4 years
Bruijn (1994)	Mild to moderate essential hypertension	170 (66/104)	48.2 ± 2.3	N/A	Parallel	N/A	Trandolapril	0.5, 1, and, 2 mg/day	4 weeks
Bull (2015)	Aortic stenosis	96 (71/25)	68.6 ± 14.1	N/A	Parallel	United Kingdom	Ramipril	2.5-10 mg/day	12 months
Burggraaf (1998)	None	9 (9/0)	N/A	N/A	Latin square design	Netherlands	Captopril	50 mg/day	1 day
Burris (1991)	Mild to moderate hypertension	100 (67/33)	N/A	N/A	Parallel	N/A	Ramipril	10 mg/day	4 weeks
Bussmann (1987)	Chronic heart failure	23 (N/A)	N/A	N/A	Parallel	N/A	Captopril	36-150 mg/day	6 months
Caletti (2011)	Post-diarrheal hemolytic uremic syndrome nephropathy	30 (12/18)	7.6 ± 3.4	N/A	Parallel	N/A	Enalapril	0.2 mg/kg/day	7 months
Campbell (1985)	None	6 (6/0)	N/A	N/A	N/A	N/A	Captopril	50 mg/day	1 day
Capek (1994)	Type 2 diabetes with microalbuminuria	15 (7/8)	63.9 ± 6.2	N/A	Parallel	N/A	Captopril	37.5 mg/day	12 months
CAPTOPRIL MULTICENTER RESEARCH GROUP (1983)	Refractory chronic congestive heart failure	92 (87/5)	N/A	N/A	Parallel	N/A	Captopril	75-150 mg/day	12 weeks
Cardillo (1994)	Mild to moderate essential hypertension	18 (15/3)	N/A	N/A	Latin-square design	N/A	Benazepril	10 and 20 mg/day	2 weeks
Carlsen (1989)	Hypertension with chronic kidney disease	27 (14/13)	N/A	N/A	N/A	Denmark	Cilazapril	0.5-5.0 mg/day	8 weeks
Carp (1994)	Healthy volunteers underwent epidural catheter	20 (6/14)	30.5 ± 7.9	N/A	Parallel	USA	Enalapril	1.25 mg IV	1 day
Carré (1992)	None	15 (15/0)	27.0 ± 5.0	N/A	Cross-over	France	Captopril	50 mg/day	1 month

Challenor (1991)	Raynaud's phenomenon	21 (2/19)	N/A	N/A	Cross-over	United Kingdom	Enalapril	20 mg/day	4 weeks
Chalmers (1987)	Congestive heart failure	130 (90/40)	N/A	Black, White, and Others	Parallel	Australia, Germany, Colombia, Belgium, New Zealand, Brazil, South Africa, Peru, and United Kingdom	Lisinopril	5-20 mg/day	12 weeks
Chatellier (1987)	Mild to moderate uncomplicated hypertension	205 (147/58)	47.0 ± 8.9	N/A	Parallel	N/A	Enalapril	20 mg/day	6 months
Chen (2002)	Angina pectoris and myocardial ischemia	20 (15/5)	67.0 ± 3.2	N/A	Parallel	N/A	Lisinopril	10 mg/day	8 weeks
Cheung (1999)	Mild essential hypertension	33 (20/13)	49.6 ± 14.2	N/A	Parallel	N/A	Fosinopril	10-20 mg/day	12 weeks
Cheung and Lau (1999)	Mild essential hypertension	33 (20/13)	49.6 ± 14.2	N/A	Parallel	N/A	Fosinopril	10-20 mg/day	12 weeks
Chinese Cardiac Study (CCS-1) (1995)	Suspected acute myocardial infarction	13634 (N/A)	N/A	N/A	Parallel	China	Captopril	6.25-37.5 mg/day	4 weeks
Chrysant (1985)	Heart failure	14 (14/0)	61.0 ± 11.5	N/A	Parallel	N/A	Enalapril	10 mg/day	14 weeks
Chrysant (1993)	Hypertension	289 (182/107)	53.4 ± 11.3	White, Black, and Others	Parallel	N/A	Perindopril	2-16 mg/day	16 weeks
Chrysant (1996)	Hypertension	82 (50/32)	N/A	White, Black, and Others	Parallel	N/A	Benazepril	5, 10, and 20 mg/day	6 weeks
Chrysant (1997)	Mild to moderate essential hypertension	208 (135/73)	N/A	N/A	Parallel	N/A	Perindopril	2, 4, and 8 mg/day	12 weeks
Chrysant (2000)	Essential hypertension	239 (134/105)	53.8 ± 10.7	White and Others	Parallel	N/A	Enalapril	5-40 mg/day	11 weeks
Circo (1995)	Congestive heart failure	101 (77/24)	62.0 ± 4.9	N/A	Parallel	N/A	Delapril	15-60 mg/day	4 weeks
Cleland (1985)	Chronic heart failure	20 (N/A)	N/A	N/A	Cross-over	N/A	Enalapril	5-40 mg/day	8 weeks

Cleland (1991)	Angina pectoris and heart failure	18 (14/4)	63.0 ± 6.0	N/A	Cross-over	N/A	Captopril	50-150 mg/day	12 weeks
Cleland (1999)	Chronic heart failure	850 (468/382)	75.3 ± 5.2	N/A	Parallel	Bulgaria, Czech Republic, Hungary, Ireland, Poland, Russia, Slovakia, and the United Kingdom	Perindopril	4 mg/day	1 years
Cockerham (2017)	HIV infection	31 (31/0)	52.1 ± 9.6	White, African-American, Latino/Hispanic, Asian, and Pacific Islander	Parallel	California	Lisinopril	20 mg/day	24 weeks
Colfer (1992)	Congestive heart failure	172 (143/29)	N/A	N/A	Parallel	N/A	Benazepril	2-20 mg/day	12 weeks
CONSENSUS Trial Study Group (1987)	Severe congestive heart failure	253 (178/75)	N/A	N/A	Parallel	Finland, Norway, and Sweden	Enalapril	10-40 mg/day	188 days
Coppo (2007)	IgA nephropathy and moderate proteinuria	66 (48/18)	20.5 ± 6.3	N/A	Parallel	Italy, France, Germany, Sweden, and Portugal	Benazapril	0.2 mg/kg/day	38 months
Corbalán (1990)	Congestive heart failure	16 (12/4)	61.3 ± 8.6	N/A	Cross-over	N/A	Captopril	150 mg/day	9 weeks
Cordonnier (1999)	Type 2 diabetes with glomerulosclerosis	22 (18/4)	47.0 ± 9.5	N/A	Parallel	France	Perindopril	4 mg/day	2 years
Costagliola (1995)	Glaucoma	40 (20/20)	N/A	N/A	Cross-over	N/A	Captopril	25 mg/day	1 week
Crepaldi (1998)	Type 1 diabetes	66 (44/22)	37.4 ± 10.4	N/A	Parallel	Italy	Lisinopril	20 mg/day	36 months
Dahlof (2005)	Hypertension	621 (357/268)	51.7 ± 10.4	N/A	Parallel	Austria, Belgium, Canada, Finland,	Enalapril	10 mg/day	12 weeks

						France, Greece, Norway, Poland, Spain, Sweden, Switzerland and, United Kingdom			
Dalsgaard (2014)	Severe aortic stenosis	44 (28/16)	70.0 ± 8.3	N/A	Parallel	N/A	Trandolapril	0.5-2 mg/day	8 weeks
Darasz (1995)	Acute myocardial infarction	47 (39/8)	53.6 ± 12.0	N/A	Parallel	Finland	Captopril	75 mg/day	6 months
DeQuattro (1997)	Primary hypertension	205 (N/A)	N/A	Caucasian, African-American, Asian, Mexican, and Others	Factorial design	United States	Trandolapril	0.5, 2, and 8 mg/day	6 weeks
DeQuattro and Lee (1997)	Hypertension	205 (N/A)	N/A	White, Black, Asian, Mexican, and Others	Parallel	N/A	Trandolapril	0.5, 2, and 8 mg/day	6 weeks
Di Marco (2010)	Chronic obstructive pulmonary disease	18 (16/2)	69.0 ± 11.0	N/A	Cross-over	N/A	Enalapril	10 mg/day	4 weeks
Dickstein (1991a)	Mild heart failure and previous myocardial infarction	41 (41/0)	64.0 ± 7.3	N/A	Parallel	Norway	Enalapril	10-20 mg/day	48 weeks
Dickstein (1991b)	Myocardial infarction with mild exercise intolerance	160 (160/0)	60.3 ± 7.6	N/A	Parallel	N/A	Enalapril	20 mg/day	48 weeks
Dicpinigaitis and Dobkin (1996)	None	20 (10/10)	N/A	N/A	Parallel	United States	Lisinopril	10-20 mg/day	2 weeks
Dössegger (1993)	Chronic heart failure	107 (72/35)	61.6 ± 11.2	N/A	Parallel	N/A	Cilazapril	1-5 mg/day	12 weeks
Drayer and Weber (1983)	Essential hypertension	294 (207/87)	N/A	White, Black, and Oriental	Parallel	United States	Captopril	50, 100, and 200 mg/day	8 weeks

Duboc (2005)	Duchenne muscular dystrophy	57 (N/A)	10.7 ± 1.2	N/A	Parallel	France	Perindopril	2-4 mg/day	5 years
Duboc (2007a)	Duchenne muscular dystrophy	57 (N/A)	10.6 ± 1.2	N/A	Parallel	France	Perindopril	2-4 mg/day	5 years
Duboc (2007b)	Duchenne muscular dystrophy	58 (N/A)	10.6 ± 1.2	N/A	Parallel	France	Perindopril	2-4 mg/day	10 years
Dyker (1997)	Recent cerebral ischemic stroke	24 (N/A)	68.9 ± 15.6	N/A	Parallel	N/A	Perindopril	4 mg/day	15 days
Ekholm (2002)	Hypertension	16 (10/6)	N/A	Caucasian	Cross-over	N/A	Ramipril	2.5-10 mg/day	6 weeks and 6 months
Erhardt (1995)	Heart failure	308 (229/79)	63.4 ± 8.5	White and Others	Parallel	Belgium, Denmark, Finland, Iceland, Israel, Netherland, Poland, and Sweden	Fosinopril	10-40 mg/day	12 weeks
Evans (1987)	Mild to moderate hypertension	14 (7/7)	N/A	Black and White	Parallel	N/A	Enalaprilat	1.25 mg IV every 6 hours	1 day
Eveson (2007)	Hypertension	40 (25/15)	74.1 ± 9.9	N/A	Parallel	United Kingdom	Lisinopril	5-10 mg/day	90 days
Fagard (2001)	Essential hypertension	9 (9/0)	46.0 ± 6.0	White	Cross-over	Belgium	Enalapril	20 mg/day	24 hours
Faxon (1995)	Coronary angioplasty	1436 (1153/283)	58.04 ± 10.04	N/A	Parallel	European countries	Cilazapril	2, 10, and 20 mg/day	6 months
Ferrari (2006)	Acute myocardial infarction	1252 (816/436)	72.5 ± 5.5	N/A	Parallel	European countries	Perindopril	8 mg/day	12 months
Flather (1994)	Acute myocardial infarction	267 (214/53)	61.5 ± 11.5	N/A	Three-way	United Kingdom	Captopril	6.25-37.5 mg/day	28 days
	Acute myocardial infarction	474 (362/112)	59.5 ± 15.2	N/A	Factorial design	United Kingdom	Captopril	6.25-100 mg/day	28 days

Ford (1993)	Mild to moderate essential hypertension	64 (19/45)	51.9 ± 10.3	Caucasian	Parallel	United States	Fosinopril	10, 20, and 40 mg/day	4 weeks
Forette (1987)	Hypertension	32 (3/29)	N/A	N/A	Parallel	N/A	Enalapril	20-40 mg/day	8 weeks
Foucan (1998)	Sickle cell anemia	22 (7/15)	29.1 ± 7.1	N/A	Parallel	France	Captopril	25 mg/day	6 months
Fox (2003)	Stable coronary artery disease	12218 (10439/1779)	60.0 ± 8.9	N/A	Parallel	United Kingdom	Perindopril	8 mg/day	4.2 years
Franciosa (1985)	Chronic congestive heart failure	17 (N/A)	56.6 ± 9.3	N/A	Parallel	N/A	Enalapril	10-20 mg/day	12 weeks
Frcka and Lader (1988)	None	12 (6/6)	N/A	N/A	Latin-square design	N/A	Enalapril	20 mg/day	8 days
French (1999)	Myocardial infarction	493 (385/108)	58.5 ± 10.0	N/A	Parallel	N/A	Captopril	6.26-150 mg/day	3 weeks
Fuller (1987)	None	6 (6/0)	N/A	N/A	N/A	N/A	Enalapril	5 mg/day	2 days
Galcera-Tomas (1993)	Acute myocardial infarction	40 (N/A)	55.0 ± 9.9	N/A	Parallel	N/A	Captopril	6.25-75 mg/day	2 weeks
Gall (1992)	Type 2 diabetes with primary hypertension	23 (13/10)	58.2 ± 8.3	N/A	Latin square design	N/A	Captopril	25-50 mg/day	8 weeks
Gansevoort (1994)	Non-diabetic proteinuria and hypertension	11 (9/2)	41.2 ± 1.8	Caucasian	N/A	N/A	Enalapril	10 and 20 mg/day	4 weeks
Gerritsen (1998)	Type 2 diabetes and hypertension	81 (54/27)	60.3 ± 8.7	N/A	Parallel	Netherlands	Enalapril	10-20 mg/day	48 weeks
Gianni (2007)	Elderly vascular disease	2755 (1,925/830)	74.1 ± 3.7	N/A	Parallel	Canada, United States, Western Europe, and South America	Ramipril	2.5-10 mg/day	4.3 ± 2.6 years
Gilbert (1993)	Heart failure	14 (10/4)	50 ± 11.2	N/A	Cross-over	United States	Lisinopril	5-20 mg/day	12 weeks
Gleerup (1996)	Mild hypertension	13 (13/0)	N/A	N/A	Cross-over	N/A	Spirapril	6 mg/day	4 weeks

Gliddon (2007)	Cutaneous systemic sclerosis with Raynaud's phenomenon (RP)	210 (31/179)	54.5 ± 12.0	Caucasian and Others	Parallel	England and Wales	Quinapril	80 mg/day	2-3 years
Gomez (1989)	Mild to moderate essential hypertension	216 (194/22)	N/A	Caucasian, Negro, and Others	Parallel	United States and Sweden	Lisinopril	1.25, 5, 20, and 80 mg/day	6 weeks
González-Fernández (1993)	Acute myocardial infarction	50 (28/22)	61.5 ± 10.7	N/A	Parallel	N/A	Enalapril	5 mg/day	4 weeks
Gordon (1997)	Hypertension	34 (34/0)	42.0 ± 6.0	N/A	Cross-over	N/A	Enalapril	10 -20 mg/day	4 weeks
Gøtzsche (1992)	Acute myocardial infarction	58 (52/6)	N/A	N/A	Parallel	N/A	Captopril	6.25-50 mg/day	6 months
Gradman (1995)	Hypertension	161 (103/58)	52.1 ± 13.6	White, Black, and Hispanic	Parallel	N/A	Enalapril	20 mg/day	8 weeks
Gross (2020)	Alport's syndrome	20 (20/0)	8.0 ± 4.5	Caucasian and Turkish	Parallel	Germany	Ramipril	1-6 mg/m^2 body surface area	3-6 years
Guazzi (1999)	Congestive heart failure	20 (16/4)	58.0 ± 8.0	N/A	Cross-over	Italy	Enalapril	20 mg/day	8 weeks
Guglin (2019)	Breast cancer	308 (N/A)	50.8 ± 10.6	Black, African American, White, Hispanic, Latino origin, and Others	Parallel	United States	Lisinopril	10 mg/day	1 year
Guillevin (1981)	Hypertension	26 (11/15)	47.0 ± 1.3	N/A	Parallel	N/A	Captopril	1 mg/kg for short term and 5.1 ± 0.6 mg/kg for long term	1 day for short term and 4 months for long term
Güntzel (1991)	Hypertension	85 (56/ 29)	48.2 ± 12.4	White	Parallel	N/A	Cilazapril	2.5 and 5 mg/day	8 weeks
Gupta (1990)	Hypertension	10 (10/0)	42.0 ± 3.0	White	Cross-over	United States	Quinapril	40 mg/day	4 weeks
Hampton (1998)	Moderate heart failure	292 (222/70)	N/A	N/A	Parallel	United Kingdom and Poland	Trandolapril	1-4 mg/day	16 weeks

Hatazawa (2004)	Previous minor stroke	19 (13/6)	65.1 ± 8.3	N/A	Parallel	N/A	Perindopril	2-4 mg/day	4 weeks
Heart Outcomes Prevention Evaluation (HOPE) Study Investigators (2000)	Diabetes mellitus	3577 (2255/1322)	N/A	N/A	Factorial design	North and South America and Europe	Ramipril	2.5 mg/day	4.5 years
Henderson (1994)	Hypertension	26 (26/0)	33.0 ± 9.2	N/A	Parallel	Australia	Captopril	12.5-100 mg/day	24 weeks
Hernández (2000)	Renal transplantation	52 (34/18)	48.7 ± 13.0	N/A	Parallel	Spain	Lisinopril	10 mg/day	12 months
Herrlin (1990)	Heart failure	19 (16/3)	51.5 ± 5.4	N/A	Parallel	N/A	Enalapril	10 mg/day	1 day
Heuvel (1997)	Myocardial infarction	244 (191/53)	59.0 ± 1.0	N/A	Parallel	N/A	Captopril	75 mg/day	12 months
Holdaway (1994)	Type 2 diabetes	55 (31/24)	55.4 ± 1.2	N/A	Parallel	New Zealand	Fosinopril	10-20 mg/day	26 weeks
Holwerda (1996)	Mild to moderate uncomplicated hypertension	211 (95/116)	52.9 ± 12.1	N/A	Parallel	Italy, Netherlands, and France	Enalapril	20 mg/day	8 weeks
Hommel (1986)	Type 1 diabetes with nephropathy	16(14/2)	31.0 ± 9.0	N/A	Parallel	Denmark	Captopril	50-100 mg/day	12 weeks
Homuth (1993)	Hypertension	160 (95/65)	47.0 ± 9.2	N/A	Parallel	Eastern Germany	Ramipril	2.5-10 mg	6 weeks
Hong (2018)	Gestational hypertension	142 (N/A)	31.3 ± 8.4	N/A	Parallel	China	Captopril	0.5 - 2.5 mg/kg/day	4 weeks
Hou (2006)	Chronic kidney disease	224 (111/113)	44.7 ± 15.4	Chinese	Parallel	China	Benazapril	20 mg/day	3.4 years
Hoy (2019)	None	125 (98/27)	N/A	N/A	Parallel	Australia	Perindopril	5-10 mg/day	60 months
Hryniewicz (2005)	Chronic heart failure	32 (28/4)	55.0 ± 2.7	N/A	Parallel	United States	Ramipril	10 mg/day	1 day
Hsu (2009)	Single ventricle	230 (161/69)	13.8 ± 9.2	White, Black, Asian, and Others	Parallel	N/A	Enalapril	0.1-0.4 mg/kg/day	14 months
Hsu (2010)	Single ventricle	230 (141/89)	20.1 ± 9.2	White, Black, and Asian	Parallel	United States and Canada	Enalapril	0.1-0.4 mg/kg/d	14 months

Hutcheon (2002)	Left ventricular systolic dysfunction	66 (33/33)	81.0 ± 5.7	N/A	Parallel	N/A	Perindopril	2-4 mg/day	10 weeks
ISIS-4 (Fourth International Study of Infarct Survival) Collaborative Group (1995)	Suspected acute myocardial infarction	58050 (N/A)	N/A	N/A	Factorial design	N/A	Captopril	12.5-100 mg/day	1 month
Janbabai (2017)	Newly diagnosed malignancy	69 (5/64)	47.4 ± 12.02	N/A	Parallel	N/A	Enalapril	10-20 mg/day	6 months
Janini (1988)	Raynaud's phenomenon	17 (3/14)	44.1 ± 17.04	N/A	Cross-over	United Kingdom	Enalapril	20 mg/day	3 weeks
Javannnard (2011)	Migraine	40 (6/34)	34.4 ± 1.8	N/A	Parallel	Iran	Enalapril	10 mg/day	2 months
Jerums (2001)	Type 1 diabetes and microalbuminuria	23 (11/12)	32.0 ± 5.5	N/A	Parallel	Australia	Perindopril	2-8 mg/day	36 months
Jirasirirojanakorn (1998)	Chronic left-sided valvular regurgitations	60 (27/33)	36.8 ± 17.7	N/A	Parallel	N/A	Captopril	50 and 100 mg/day	4 weeks
Jones-Burton (2010)	Hypertension	97 (49/48)	52.3 ± 7.6	Black, White, Hispanic, Latino, and Others	Parallel	N/A	Enalapril	20 mg/day	4 weeks
Jose (2006)	Systolic dysfunction after myocardial infarction	1854 (1544/310)	59.0 ± 10.6	N/A	Parallel	N/A	Captopril	37.5-75 mg/day	2 weeks
Joy (1987)	Congestive heart failure	55 (43/12)	N/A	N/A	Parallel	United Kingdom	Lisinopril	10-20 mg/day	24 weeks
Kaski (1994)	Syndrome X with microvascular angina	10 (3/7)	53.0 ± 6.0	N/A	Cross-over	N/A	Enalapril	10 mg/day	2 weeks
Katayama (2002)	Type 1 diabetes	79 (28/51)	57.3 ± 8.2	Japanese	Parallel	Japan	Captopril and Imidapril	37.5 mg/day and 5 mg/day	1.48 years
Katz (1991)	None	34 (22/12)	N/A	N/A	Parallel	United States	Enalapril and Captopril	20 and 75 mg/day	7 days

Kaufman (1993)	None	20 (10/10)	39.0 ± 9.0	N/A	Cross-over	United States	Enalapril	20 mg/day	5 days
Kim (2015)	Orthognathic surgery	48 (26/22)	22.1 ± 3.7	N/A	Parallel	Korea	Enalapril	10 mg/day	1 day
Kitzman (2010)	Heart failure with preserved ejection fraction	71 (11/60)	69.5 ± 7.5	Black and Others	Parallel	United States	Enalapril	5-20 mg/day	12 months
Kjekshus and Swedberg (1988)	Congestive heart failure	253 (N/A)	N/A	N/A	Parallel	N/A	Enalapril	2.5-40 mg/day	20 months
Kjekshus and Swedberg (1989)	Congestive heart failure	253 (179/74)	N/A	N/A	Parallel	Finland, Norway, and Sweden	Enalapril	10-40 mg/day	6 months
Klapholz (2001)	Heart Failure	113 (97/16)	56.9 ± 11.7	N/A	Parallel	N/A	Omapatrilat	1, 2.5, 5, 10, 25, and 50 mg/day	2 days
Kleber (1992)	Mild heart failure	170 (128/42)	N/A	N/A	Parallel	Germany	Captopril	12.5-50 mg/day	2.7 years
Kloke (1990)	Hypertension with chronic kidney failure	11 (8/3)	46.0 ± 9.3	N/A	N/A	N/A	Cilazapril	1.25-2.5 mg/day	1 week
Kondowe (1987)	None	10 (10/0)	N/A	N/A	Cross-over	N/A	Captopril	37.5 mg/day	2 weeks
Konstam (1992)	Heart failure	56 (41/15)	60.3 ± 9.1	N/A	Parallel	Boston, Tenn, and Belgium	Enalapril	10-20 mg/day	1 year
Konstam (1993)	Asymptomatic systolic dysfunction	157 (125/30)	N/A	N/A	Parallel	Boston, Tenn, and Belgium	Enalapril	10-20 mg/day	1 year
Kontopoulos (1999)	Acute myocardial infarction	60 (53/7)	N/A	N/A	Parallel	N/A	Quinapril	5-10 mg/day	1 month
Koskinen (1988)	Mild to moderate hypertension	23 (11/12)	N/A	N/A	Parallel	N/A	Quinapril	20-80 mg/day	12 weeks
Kouatli (1997)	Patients who underwent the Fontan procedure	18 (N/A)	14.5 ± 6.2	N/A	Cross-over	United States	Enalapril	15 mg/day	10 weeks
Krysiak and Okopien (2008)	Stable coronary artery disease	76 (61/15)	52.6 ± 8.6	N/A	Parallel	Poland	Enalapril and Perindopril	20 and 4 mg/day	90 days

Krysiak and Okopien (2011)	Coronary artery disease	174 (105/29)	51.4 ± 5.3	N/A	Parallel	N/A	Enalapril and Perindopril	20 and 4 mg/day	90 days
Küppers (1997)	Mild to moderate essential hypertension	92 (48/44)	52.4 ± 10.0	N/A	Parallel	Germany	Enalapril	5-10 mg/day	8 weeks
Kuschnir (1996)	Hypertension	154 (66/88)	56.5 ± 9.1	White	Parallel	N/A	Benazepril	20 mg/day	8 weeks
Kvetny (2001)	Type 1 diabetes	89 (51/38)	45.0 ± 13.5	N/A	Parallel	N/A	Perindopril	4 mg/day	36 months
Labinjoh (2001)	None	8 (8/0)	N/A	N/A	Cross-over	N/A	Quinapril	40 mg/day	7 days
Lang (1995)	None	12 (7/5)	72.0 ± 6.9	N/A	Cross-over	United Kingdom	Lisinopril	2.5-10 mg/day	5 weeks
Laurent (2015)	Mild to moderate uncomplicated hypertension	795 (373/422)	51.6 ± 11.5	N/A	Parallel	France, Russia, Ukraine, Latvia, Lithuania, and Hungary	Perindopril	3.5 and 5 mg/day	8 weeks
Leary (1985)	None	13 (13/0)	N/A	N/A	N/A	N/A	Captopril	100 mg/day	1 days
Lechat (1993)	Congestive heart failure	125 (94/31)	59.4 ± 7.8	N/A	Parallel	France and United Kingdom	Perindopril	2-4 mg/day	3 months
Lee (1999)	Hyperlipidemia	40 (29/11)	48.8 ± 8.7	N/A	Parallel	N/A	Lisinopril	20 mg/day	6 months
Lees and Reid (1987)	None	36 (36/0)	N/A	N/A	Parallel	Scotland	Perindopril	1, 2, 4, 8, and 16 mg/day	1 weeks
Leonetti (1991)	Elderly	36 (10/26)	N/A	N/A	Latin-square design	N/A	Captopril	50 mg/day	2 months
Lerch (1999)	Hypertension with type 2 diabetes	30 (21/9)	57.1 ± 7.2	White	Parallel	Switzerland	Temocapril	20 mg/day	6 weeks
Levine and Applegate (1997)	Mild to moderate hypertension	321 (N/A)	N/A	Black and Non-black	Parallel	N/A	Trandolapril	0.5-8 mg/day	6 weeks
Lewis (1988)	Congestive heart failure	130 (N/A)	N/A	N/A	Parallel	Australia, Germany, Colombia, Belgium,	Lisinopril	5-20 mg/day	12 weeks

						New Zealand, Brazil, South Africa, Peru, and United Kingdom			
Lewis (1993)	Diabetic nephropathy	409 (207/202)	34.5 ± 7.5	Black and White	Parallel	N/A	Captopril	75 mg/day	12 months
Lewis (1996)	Diastolic hypertension	369 (221/148)	N/A	African-American and White	Parallel	United States	Enalapril	5 mg/day	4 years
Liao (2003)	None	63 (63/0)	32.0 ± 8.0	N/A	N/A	United States	Omapatrilat	10, 25, 50, 75, and 125 mg/day	10 days
Lonn (2001)	Atherosclerosis progression	732 (607/125)	65.4 ± 5.3	N/A	Factorial design	Canada	Ramipril	2.5 and 10 mg/day	4.5 years
Lonn (2002)	High risk of cardiovascular events	2,480 (0/2,480)	66.7 ± 6.9	N/A	Factorial design	N/A	Ramipril	2.5-10 mg/day	4.5 years
Lonn (2009)	Impaired glucose tolerance or impaired fasting glucose	1,425 (655/770)	54.3 ± 10.9	N/A	Factorial design	Hamilton, Ontario, Canada, Rosario, Argentina, Chennai, India, and Quebec City, Quebec, and Canada	Ramipril	5-15 mg/day	3 years
Lough (1998)	Mild to moderate heart failure	178 (N/A)	N/A	N/A	Parallel	United kingdom	Quinapril	5-20 mg/day	1 week
Luca (1993)	Hypertension	28 (21/7)	45.0 ± 5.1	N/A	Parallel	Italy	Benazapril	10-20 mg/day	6 weeks
Luksch (2006)	None	12 (12/0)	28.3 ± 2.7	N/A	Cross-over	Austria	Enalapril	4 mg IV	1 day
MacDonald (1990)	None	30 (30/0)	24.0 ± 4.6	N/A	Parallel	N/A	Zabicipril	0.25, 0.5, 1, 2.5, and 10 mg/day	96 hours
MacFadyen (1997)	Heart failure	23 (17/6)	67.0 ± 8.0	Caucasian	Parallel	N/A	Perindopril	2-4 mg/day	8 weeks

MacFadyen and Struthers (1997)	None	16 (16/0)	N/A	N/A	Latin square design	N/A	Lisinopril	20 mg/day	7 days
Maclean (1989)	Essential hypertension	270 (181/89)	N/A	White, Black, and Asian	Parallel	N/A	Quinapril	20-80 mg/day	12 weeks
MacMahon (2000)	Occlusive arterial disease	617 (506/111)	60.5 ± 8.0	N/A	Parallel	New Zealand	Ramipril	5-10 mg/day	2-4 years
Malik (1998)	Type 1 or type 2 diabetes and mild neuropathy	41 (41/0)	48.4 ± 11.1	N/A	Parallel	United Kingdom	Trandolapril	2 mg/day	12 months
Mancia (1992)	Mild to moderate essential hypertension	62 (46/16)	51.3 ± 9.0	N/A	Parallel	N/A	Trandolapril	2 mg/day	6 weeks
Mancia (2014)	Hypertension	340 (166/174)	53.4 ± 9.8	White	Parallel	France, Germany, Italy, Poland, Russia, Spain, and Ukraine	Enalapril	10 and 20 mg/day	10 weeks
Mandelbrot (2015)	Kidney transplantation	295 (197/98)	47.1 ± 12.7	White, Black, Asian, and Others	Parallel	North America, Latin America, Europe, Middle East, Australia, and Africa	Ramipril	5-10 mg/day	52 weeks
Marketou (2008)	Type 2 diabetes	62 (33/29)	63.5 ± 8.4	N/A	Parallel	N/A	Perindopril	4 mg/day	6 months
Marney (2010)	Metabolic syndrome	24 (12/12)	42.8 ± 2.2	Black, White, and Hispanic	Cross-over	United States	Enalapril	5 and 10 mg/day	5 days
Marre (2004)	Type 2 diabetes and microalbuminuria or proteinuria	4912 (3432/1480)	65.1 ± 8.3	N/A	Parallel	European and North African , Austria, Belgium, Croatia, Czech Republic, France,	Ramipril	1.25 mg/day	3 years

						Germany, Greece, Hungary, Morocco, Netherlands, Slovenia, Spain, Switzerland, Tunisia, Turkey, and United Kingdom			
Martinuzzi (2008)	McArdle's disease	8 (6/2)	34.3 ± 10.5	N/A	Cross-over	N/A	Ramipril	2.5 mg/day	12 weeks
Maschio (1996)	Chronic renal insufficiency	583 (421/162)	51.0 ± 12.5	N/A	Parallel	Italy, Germany, and France	Benazepril	10 mg/day	3 years
Mathur (2020)	Single ventricle	230 (N/A)	22.0 ± 12.3	White, Black, Asian, Hispanic, and Others	Parallel	United States and Canada	Enalapril	0.10-0.38 mg/kg/day	14 months
Matsumoto (2006)	End-stage renal disease undergoing maintenance hemodialysis	27 (15/12)	54.3 ± 12.9	N/A	Parallel	N/A	Imidapril	2.5 mg/day	6 months
Mauer (2009)	Type 1 diabetes	189 (88/101)	29.9 ± 9.6	White and Others	Parallel	N/A	Enalapril	20 mg/day	5 years
Mazayev (1998)	Heart failure	41 (33/8)	55.3 ± 12.4	Caucasian and Others	Parallel	N/A	Lisinopril	5-10 mg/day	28 days
Mbah (2012)	Idiopathic oligospermia and infertility	33 (33/0)	28.9 ± 8.2	N/A	Cross-over	Nigeria	Lisinopril	2.5 mg/day	5 years
McNabb (1986)	None	8 (8/0)	N/A	Caucasian	Cross-over	United Kingdom	Enalapril	20 mg/day	2 weeks
Mersey (1993)	Mild to moderate hypertension	129 (71/58)	n/a	White	Parallel	N/A	Captopril	25 mg/day	8 weeks
Messerli (1998)	Hypertension	311 (209/102)	N/A	Black, White, and Oriental	Parallel	N/A	Trandolapril	4 mg/day	6 weeks
Metelitsa (1992)	Stable angina pectoris	14 (14/0)	N/A	N/A	N/A	Russia	Captopril	50-100 mg/day	1 day

Miller (1994)	Renal dysfunction and hypertension	48 (28/20)	50.2 ± 13.1	White and Black	Parallel	N/A	Quinapril	2.5-40 mg/day	4 weeks
Mitrovic (1996)	Heart failure	50 (41/9)	N/A	N/A	Parallel	Germany	Quinapril	0.5, 1, 2.5, 5, 10 mg IV	1 day
Morgan (1995)	Essential hypertension	17 (17/0)	67.5 ± 3.4	N/A	Cross-over	N/A	Enalapril and Perindopril	5, 10, 20, 40 mg/day of enalapril and 2, 4, 8 mg/day of perindopril	2 weeks
Moser (1991)	Mild to moderate essential hypertension	173 (114/59)	N/A	White, Blacks, Hispanic, and Others	Parallel	N/A	Benazepril	2, 5, 10, and 20 mg/day	4 weeks
Moser and Lunn (1982)	Hypertension	30 (N/A)	N/A	Black	Parallel	N/A	Captopril	450 mg/day	3 months
Moyses and Higgins (1992)	Congestive heart failure	468 (N/A)	N/A	Caucasian, Black, and Others	Parallel	N/A	Lisinopril	N/A	3 months
Muiesan (1993)	Chronic stable angina pectoris	24 (21/3)	N/A	N/A	Cross-over	Italy	Benazapril	20 mg/day	1 week
Mullen (1998)	Type 1 diabetes	91 (56/35)	30.8 ± 6.4	N/A	Parallel	United Kingdom	Enalapril	5-20 mg/day	24 weeks
Mulligan (1988)	Congestive heart failure	16 (14/2)	62.0 ± 11.0	N/A	Cross-over	N/A	Enalapril	10 mg/day	6 weeks
Nabel (1991)	Acute myocardial infarction	38 (31/7)	54.6 ± 11.5	N/A	Parallel	United States	Captopril	2-8 mg IV and oral 25-100 mg/day	4 hours injection followed 3 months oral phase
Naeije (1993)	None	16 (16/0)	N/A	N/A	N/A	N/A	Zabicipril	0.5, 1, and 2.5 mg/day	1 weeks
Nagamia (2007)	Metabolic syndrome	44 (24/20)	N/A	N/A	Cross-over	United States	Quinapril	20 mg/day	24 weeks
Nalbantgil (1998)	Syndrome X	18 (15/3)	43.2 ± 4.6	N/A	Cross-over	N/A	Cilazapril	5 mg/day	3 weeks
Naranjo (1991)	Alcoholism	42 (36/6)	42.1 ± 9.6	N/A	Cross-over	Canada	Enalapril	10 and 20 mg/day	4 weeks
Nazir (2005)	Mild early ischemic stroke	25 (18/7)	69.8 ± 10.9	N/A	Parallel	United Kingdom	Perindopril	4 mg/day	2 weeks

Neaton (1993)	Mild hypertension	369 (221/148)	N/A	Black and Non-black	Parallel	United States	Enalapril	5 mg/day	18 months
Nicholls (1984)	Heart failure	16 (N/A)	N/A	N/A	Parallel	New Zealand	Enalapril	10 mg/day	12 weeks
Nicolas (2012)	None	28 (28/0)	N/A	N/A	Parallel	N/A	Enalapril	20 mg/day	1 day
Northridge (1993)	Chronic heart failure	32 (30/2)	N/A	Caucasian and Others	Cross-over	United Kingdom	Quinapril	2.5-20 mg/day	8 weeks
Northridge (1999)	Chronic heart failure	38 (30/8)	N/A	N/A	Parallel	United Kingdom	Captopril	25-100 mg/day	12 weeks
O'Hare (2000)	Type 1 diabetes	134 (95/39)	40.0 ± 11.9	N/A	Parallel	United Kingdom and Ireland	Ramipril	1.25 and 5 mg/day	2 years
O'Neill (1992)	Heart failure	20 (8/12)	81.0 ± 6.0	N/A	Cross-over	N/A	Captopril	6.25-100 mg/day	6 weeks
Olajide and Lader (1985)	None	12 (N/A)	N/A	N/A	Cross-over	N/A	Enalapril	20 mg/day	14 days
Oldroyd (1991)	Acute myocardial infarction	99 (82/17)	59.9 ± 7.1	N/A	Parallel	N/A	Captopril	6.25-75 mg/day	2 months
Oosterga (2001)	Coronary artery bypass grafting	148 (26/23)	61.9 ± 9.02	N/A	Parallel	Netherlands	Quinapril	40 mg/day	4 weeks
Oparil (1999)	Mild to moderate hypertension	90 (44/46)	N/A	Caucasian, Black, Oriental, and Others	Parallel	United States	Enalapril	20 mg/day	6 weeks
Ordaz-Medina (2010)	Hemodialysis	25 (13/12)	41.1 ± 14.0	N/A	Parallel	Mexico	Enalapril	20 mg/day	3 months
Ormesher (2020)	Preeclampsia	60 (0/60)	32.7 ± 6.6	White, Black, Asian, and Others	Parallel	United Kingdom	Enalapril	5-20 mg/day	6 months
Oshima (1997)	Acute myocardial infarction	40 (26/14)	66.9 ± 2.4	N/A	Parallel	N/A	Imidapril	2.5-10 mg/day	2 weeks
Parati (2014)	Hypertension	795 (373/422)	51.6 ± 11.4	N/A	Parallel	France, Russia, Ukraine, Latvia, Lithuania, and Hungary	Perindopril	3.5 and 5 mg/day	8 weeks
Pasquale (1993)	Cardioplegia due to aorto-coronary bypass	54 (45/9)	58.9 ± 2.8	N/A	Parallel	N/A	Captopril	4 mg/l	1 day

Paster (1998)	A history of cough induced by angiotensin-converting enzyme inhibitors	100 (51/49)	N/A	White, Oriental, Black, and Others	Parallel	United States	Lisinopril	20, and 50 mg/day	8 weeks
Patat (1989)	None	90 (90/0)	N/A	N/A	Parallel	N/A	Trandolapril	0.125, 0.25, 0.5, 1, 2, 4, 8, 16, 24, and 32 mg/day	8 days
Patel (1996)	Asymptomatic carotid artery occlusive disease	15 (9/6)	69.1 ± 6.6	White and Black	Parallel	United States	Ramipril and Enalapril	5 mg/day and 5 mg/day	1 day
Pauly (2011)	Symptoms of cardiac ischemia, nonobstructive coronary arteries, and microvascular dysfunction	61 (0/61)	54.0 ± 10.0	N/A	Parallel	N/A	Quinapril	40-80 mg/day	16 weeks
Pepine (2003)	Coronary artery disease with stable angina	336 (267/69)	64.5 ± 8.8	White and Others	Parallel	N/A	Quinapril	40-80 mg/day	16 weeks
Perez-Vasquez (2020)	Rheumatoid arthritis	53 (0/53)	49.0 ± 10.4	N/A	Parallel	Mexico	Enalapril	10 mg/day	12 weeks
Persson and Stimpel (1996)	Hypertension	151 (85/66)	N/A	Caucasian	Parallel	N/A	Moexipril	7.5 and 15 mg/day	8 weeks
Petrie (2000)	Hypertension	16 (15/1)	58.0 ± 10.6	Caucasian and Others	Cross-over	United Kingdom	Trandolapril	2 mg/day	4 weeks
Pfeffer (1988)	Myocardial infraction	59 (54/5)	57.6 ± 10.8	N/A	Parallel	N/A	Captopril	75-150 mg/day	12 months
Pfeffer (1992)	Acute myocardial infraction and left ventricular dysfunction	2231 (1,840/391)	N/A	N/A	Parallel	United States and Canada	Captopril	37.5-150 mg/day	42 ± 10 months
Pitt (2001)	Ischemic heart disease and preserved left ventricular function	1750 (1419/331)	N/A	White and Others	Parallel	United States, Canada, and Europe	Quinapril	20 mg/day	27 ± 0.3 months

Pittrow (1997)	Hypertension	130 (71/59)	N/A	Caucasian	Parallel	Germany	Spirapril	3 and 6 mg/day	12 weeks
Pituskin (2017)	Human epidermal growth factor receptor 2–overexpressing-positive early breast cancer	63 (N/A)	50.5 ± 7.5	N/A	Parallel	Canada	Perindopril	2-8 mg/day	3 weeks
Pool (1997)	Hypertension	115 (N/A)	N/A	White, Black, and Asian	Factorial design	United States	Fosinopril	5, 12.5, and 37.5 mg/day	12-13 weeks
Pool (2001)	Hypertension	231 (N/A)	N/A	White and Others	Parallel	N/A	Benazepril	10 mg/day	8 weeks
Pouleur (1992)	Left ventricular dysfunction	2569 (1926/643)	N/A	N/A	Parallel	United States, Canada, and Belgium	Enalapril	20 mg/day	4 years
Pouleur (1993)	Depressed ejection fraction	65 (54/11)	55.9 ± 9.1	N/A	Parallel	Belgium	Enalapril	5-20 mg/day	1 year
Prichard (2002)	Mild to moderate essential hypertension	103 (64/39)	52.9 ± 9.5	N/A	Parallel	Germany	Enalapril	5-20 mg/day	8 weeks
Prisant (1998)	Hypertension	234 (151/83)	N/A	Black and Non-black	Parallel	N/A	Enalapril	5, 10, 20, and 40 mg/day	12 weeks
Prisant (1999)	Hypertension	234 (151/83)	N/A	Black and Non-black	Parallel	N/A	Enalapril	5, 10, 20, and 40 mg/day	12 weeks
PROGRESS Collaborative Group (2001)	Previous stroke or transient ischemic attack	6105 (4253/1852)	64.0 ± 10.0	N/A	Parallel	Asia, Australasia, and Europe	Perindopril	4 mg/day	4 years
Puig (2007)	Hypertension	75 (40/35)	65.8 ± 5.1	N/A	Cross-over	Spain and Finland	Enalapril	20 mg/day	4 weeks
Rahman (2007)	Type 2 diabetes and impaired glucose tolerance	21 (N/A)	46.1 ± 8.2	N/A	Parallel	Malaysia	Ramipril	5 mg/day	1 year
Rahman (2010)	Type 2 diabetes	22 (N/A)	N/A	N/A	Parallel	Malaysia	Ramipril	5 mg/day	1 year
Rake (2001)	A history of angiotensin-converting enzyme inhibitor cough	132 (42/44)	N/A	N/A	Parallel	United Kingdom	Enalapril	20 mg/day	6 weeks

Ratnasabapathy (2003)	A history of cerebrovascular disease	6105 (N/A)	N/A	N/A	Parallel	Australia, New Zealand, China, France, Belgium, Italy, Japan, Sweden, United Kingdom, and Ireland	Perindopril	4 mg/day	3.9 years
Ravid (1993)	Type 2 diabetes	94 (42/52)	44.1 ± 3.3	N/A	Parallel	Israel	Enalapril	10 mg/day	5 years
Ravid (1998)	Type 2 diabetes	156 (75/81)	54.9 ± 3.0	N/A	Parallel	N/A	Enalapril	10 mg/day	6 years
Ray-Chaudhuri (1993)	None	12 (4/8)	N/A	N/A	N/A	United Kingdom	Captopril	50 mg/day	1 day
Reisin (1997)	Obesity-associated hypertension	156 (87/69)	51.5 ± 10.8	White, Black, and Other	Parallel	N/A	Lisinopril	10-40 mg/day	12 weeks
Richer (1987)	None	6 (6/0)	N/A	N/A	Cross-over	N/A	Perindopril	4, 8, and 16 mg/day	1 day
Riegger (1990)	Congestive heart failure	225 (91/134)	N/A	N/A	Parallel	N/A	Quinapril	10, 20, and 40 mg/day	12 weeks
Riegger (1991)	Mild to moderate heart failure	225 (91/134)	N/A	N/A	Parallel	N/A	Quinapril	10, 20, and 40 mg/day	3 months
Rodrigues (2008)	Hypertension	104 (51/53)	63.0 ± 11.0	N/A	Parallel	N/A	Captopril	25 mg/day	15 minutes
Rodriguez-Granillo (2007)	Coronary artery disease	118 (100/18)	56.5 ± 8.9	N/A	Parallel	N/A	Perindopril	8 mg/day	3 years
Rosenbaek (2018)	None	16 (5/11)	23.0 ± 7.5	N/A	Cross-over	Denmark	Enalapril	10 mg/day	4 days
Rouleau (2008)	Left ventricular ejection fraction	2553 (2229, 324)	61.0 ± 9.9	White and Others	Parallel	Canada, Netherlands, Belgium, and France	Quinapril	40 mg/day	43 months
Rubin (1984)	None	9 (9/0)	N/A	N/A	Latin square design	Scotland	Captopril	50 mg/day	1 day
Ruggenenti (2004)	Type 2 diabetes	601 (306/295)	62.1 ± 8.1	N/A	Parallel	Italy	Trandolapril	2 mg/day	3 years

Ruggenenti (2011)	Hypertension with type 2 diabetes	254 (170/84)	61.1 ± 7.6	White	Parallel	Italy and Slovenia	Delapril	30 mg/day	3 years
Ruilope (2004)	Type 2 diabetes	249 (143/106)	59.9 ± 9.4	N/A	Parallel	N/A	Trandolapril	2 mg/day	16 weeks
Rustin (1987)	Raynaud's phenomenon	15 (0/15)	35.7 ± 8.9	N/A	Cross-over	N/A	Captopril	75 mg/day	6 weeks
Rutledge (1988)	Moderate or severe hypertension	54 (28/26)	47.3 ± 14.62	Black, Hispanic, and White	Parallel	N/A	Enalaprilat	1.25 mg IV every 6 hours	1 day
Saito (1990)	Essential hypertension	30 (30/0)	22.0 ± 1.6	N/A	Parallel	N/A	Captopril	25, 50, and 100 mg/day	1 day
Sampaio (2005)	Chronic mitral regurgitation	47 (27/20)	39.0 ± 15.0	N/A	Parallel	N/A	Enalapril	10-40 mg/day	12 month
Sassano (1987)	Mild to moderate hypertension	205 (141/64)	47.0 ± 9.0	Black and Others	Parallel	N/A	Enalapril	20 mg/day	6 months
Saul (2013)	Borderline blood pressure	44 (31/13)	51.9 ± 11.5	N/A	Parallel	United States	Lisinopril	10-20 mg/day	10 months
Schill (1994)	Male infertility	54 (54/0)	N/A	N/A	Parallel	Germany	Captopril	50 mg/day	12 weeks
Schmidt (1992)	None	16 (16/0)	25.3 ± 6.4	N/A	Cross-over	Germany	Cilazapril	2.5 mg/day	7 days
Schneider (1999)	None	18 (14/4)	59.4 ± 2.9	N/A	Parallel	N/A	Quinaprilat	10 mg IV	1 day
Schrader (2001)	Migraine	47 (9/38)	41.3 ± 8.3	N/A	Cross-over	N/A	Lisinopril	10 mg/day	12 weeks
Seneviratne (1994)	Chronic stable heart failure	23 (N/A)	71.8 ± 6.3	N/A	Parallel	N/A	Captopril	25, 50, and 100 mg/day	12 Weeks
Shahin (2013)	Intermittent claudication	33 (25/8)	64.6 ± 7.8	N/A	Parallel	United Kingdom	Ramipril	2.5-10 mg/day	24 weeks
Sharifipour (2016)	Diabetic macular ischemia	70 (41/29)	58.0 ± 8.5	N/A	Parallel	Iran	Enalapril	5 mg/day	3 months
Sharpe (1984)	Chronic heart failure	36 (32/4)	60.5 ± 8.8	N/A	Parallel	New Zealand	Enalapril	10 mg/day	3 months
Shaw (2014)	Acute stroke	14 (7/7)	71.2 ± 7.2	N/A	Parallel	United Kingdom	Lisinopril	5-10 mg/day	7 days
Sigurdsson (1994)	Mild or moderately severe congestive heart failure	215 (N/A)	N/A	N/A	Parallel	Denmark, Finland, Norway, and Sweden	Ramipril	1.25-10 mg/day	12 weeks

Silber (2004)	Cancer with cardiac abnormality	135 (78/57)	9.5 ± 5.4	White	Parallel	N/A	Enalapril	0.05-0.15 mg/kg/d	2.80 years
Simon (1989)	Chronic stable angina	12 (11/1)	N/A	N/A	Cross-over	London	Enalapril	10-20 mg/day	2 weeks
Soejima (1996)	Acute myocardial infarction	22 (17/5)	60.0 ± 2.8	N/A	Parallel	N/A	Enalapril	5 mg/day	4 weeks
Sonbolestan (2013)	Migraine	40 (6/34)	34.4 ± 1.8	N/A	Parallel	Iran	Enalapril	10 mg/day	2 months
Speel (2005)	Atherosclerotic erectile dysfunction	51 (51/0)	60.0 ± 6.8	N/A	Parallel	Netherlands	Quinapril	20 mg/day	26-46 weeks
Squire (1994)	Heart failure	24 (11/13)	72.3 ± 9.8	N/A	Parallel	N/A	Quinapril	2.5 mg/day	1 day
Steinman (1993)	Ventricular dysrhythmia	11 (11/0)	N/A	N/A	Cross-over	N/A	Captopril	100 mg/day	6 days
Stephan (1996)	Hypertension	20 (12/8)	55.9 ± 8.9	N/A	Parallel	N/A	Ramipril	2.5-5 mg/day	15 days
Stimpel (1996)	Moderate to severe hypertension	200 (128/72)	N/A	White and Black	Parallel	Norway, Finland, United Kingdom, and Netherlands	Moexipril	3.75, 7.5, and 15 mg/day	8 weeks
Strozzi (1987)	Stable angina pectoris	12 (7/5)	N/A	N/A	N/A	N/A	Captopril	50 mg/day	1 day
Sudoh (1993)	None	8 (8/0)	N/A	N/A	Cross-over	Japan	Lisinopril	20 mg/day	1 day
Sullivan (1979)	Essential hypertension and renovascular hypertension	15 (6/9)	N/A	Black and White	Parallel	United States	Captopril	Ascending dose (10-1000 mg/day)	3 days to 12 months
Sullivan (1988)	Essential hypertension	11 (11/0)	55.0 ± 9.0	N/A	N/A	N/A	Fosinopril	20-40 mg/day	8 weeks
Sumukadas (2007)	Functional impairment	95 (29/66)	78.4 ± 6.5	N/A	Parallel	Scotland	Perindopril	2-4 mg/day	20 weeks
Sumukadas (2018)	A history of falls	80 (60/20)	78.1 ± 7.4	N/A	Parallel	Scotland	Perindopril	4 mg/day	15 weeks

Swedberg (1990)	Heart failure	253 (N/A)	N/A	N/A	Parallel	N/A	Enalapril	2.5-40 mg/day	6 months
Taddei (1995)	Essential hypertension	6 (5/1)	46.3 ± 8.3	N/A	Cross-over	N/A	Idrapril	200 mg/day	1 day
Taylor (1990)	Mild to moderate hypertension	300 (N/A)	N/A	N/A	Parallel	Europe, United States, West Germany, and France	Quinapril	10-80 mg/day	6 months - 3 years
Teo (2000)	None	460 (410/50)	61.0 ± 9.6	N/A	Factorial design	N/A	Enalapril	5-20 mg/day	47.8 months
The Acute Infarction Ramipril Efficacy (AIRE) Study Investigators (1993)	Acute myocardial infarction	1,986 (1,461/525)	60.0 ± 10.8	N/A	Parallel	Argentina, Austria, Belgium, Denmark, Finland, Germany, Ireland, Italy, Luxembourg, Netherlands, South Africa, Sweden, Switzerland, and United Kingdom	Ramipril	2.5-10 mg/day	15 months
The EUCLID study group (1997)	Type 1 diabetes and normoalbuminuria or microalbuminuria	530 (322/208)	33.7 ± 9.6	N/A	Parallel	European centres	Lisinopril	10-20 mg/day	24 months
The GISEN Group (Gruppo Italiano di Studi Epidemiologici in Nefrologia) (1997)	Glomerular filtration rate and risk of terminal renal failure in proteinuric, non-diabetic nephropathy	166 (130/36)	49.3 ± 13.6	N/A	Parallel	N/A	Ramipril	1.25-5.0 mg/day	1 months
The Treatment of Mild Hypertension	Mild hypertension	369 (221/148)	N/A	Black and Non-black	Parallel	N/A	Enalapril	2.5 mg/day	12 months

Research Group (1991)									
The Veratran Study Group (1997)	Mild and moderate essential hypertension	101 (N/A)	52.0 ± 8.9	N/A	Parallel	N/A	Trandolapril	1 mg/day	8 weeks
Thind (1985)	Moderate to severe essential hypertension	32 (13/19)	47.6 ± 13.5	Black, Caucasians, and Asian	N/A	N/A	Enalapril and Captopril	10-40 mg/day and 75-300 mg/day	1 year
Thürig (1995)	Hypertension	27 (17/10)	N/A	Caucasian	Cross-over	N/A	Lisinopril	20 mg/day	8 weeks
Tohmo (1993)	Subjected to craniotomy and ligation of an intracranial aneurysm or extirpation of an arteriovenous malformation	22 (12/10)	47.0 ± 11.8	N/A	Parallel	Finland	Enalapril	0.12 ± 0.01 mg/kg	1 day
Tong (2006)	Type 2 diabetes with moderate renal impairment	38 (25/13)	65.7 ± 5.9	N/A	Parallel	Hong Kong	Fosinopril	10-20 mg/day	2 years
Toto (1996)	Proteinuria	15 (9/6)	37.0 ± 11.3	N/A	Cross-over	United States	Ramipril	1.25 and 50 mg/day	48 weeks
Trevisan and Tiengo (1995)	Type 2 diabetes with microalbuminuria	122 (94/28)	57.01 ± 7.04	N/A	Parallel	Italy	Ramipril	1.25 mg/day	6 months
Tsang (2006)	Isolated diastolic dysfunction	21 (10/11)	68.2 ± 8.7	N/A	Parallel	United States	Quinapril	10-60 mg/day	12 months
Tuininga (2000)	Myocardial infarction	66 (N/A)	N/A	N/A	Parallel	N/A	Perindopril	4 mg/day	3 months
Turner (2006)	Intracranial aneurysm	19 (8/11)	N/A	N/A	Cross-over	United Kingdom	Perindopril	2-4 mg/day	4 weeks
Tzourio (2003)	Cerebrovascular disease	6105 (4273/1832)	64.0 ± 10.0	Asian and Others	Parallel	N/A	Perindopril	4 mg/day	3.9 years
van Kooij (2006)	Inflammatory cystoid macular edema	40 (16/24)	54.6 ± 12.3	N/A	Cross-over	Netherlands	Lisinopril	10 mg/day	3 months

Veldhuisen (1998)	Heart failure	244 (188/56)	61.2 ± 8.9	N/A	Parallel	Netherlands, Germany, and Belgium	Imidapril	2.5, 5, and 10 mg/day	12 weeks
Verrico (2016)	None	59 (46/13)	36.0 ± 9.2	African-American, Asian, Caucasian, Hispanic, and Middle eastern	Parallel	United States	Perindopril	4, 8, and 16 mg/day	10 days
Veterans Administration Co-Operative Study Group on Antihypertensive Agents (1982)	Ambulatory male veterans	399 (399/0)	N/A	White and Black	Parallel	N/A	Captopril	37.5, 75, 112.5, and 150 mg/day	7 weeks
Veterans Administration Cooperative Study Group on Antihypertensive Agents (1984)	Mild to moderate hypertension	427 (427/0)	55.2 ± 8.4	White, Black, and Others	Parallel	N/A	Captopril	37.5, 75, 112.5, and 150 mg/day	7 weeks
Viberti (1994)	Type 1 diabetes with persistent microalbuminuria	92 (51/41)	33.6 ± 9.9	N/A	Parallel	Asian and European	Captopril	100 mg/day	2 years
Vlasses (1982)	Mild to moderate essential hypertension	16 (10/6)	49.4 ± 9.2	N/A	N/A	United States	Captopril	75 mg/day	4 weeks
Vongterapak (1998)	Type 2 diabetes with microalbuminuria	28 (19/9)	46.5 ± 13.9	N/A	Parallel	Thailand	Ramipril	1.25 mg/day	12 weeks
Waeber (1999)	Essential hypertension	621 (357/268)	51.7 ± 10.4	N/A	Parallel	United Kingdom, Belgium, Sweden, France, Canada, Norway, Poland, Spain, Greece,	Enalapril	10 mg/day	12 weeks

						Switzerland, Finland, and Austria			
Walter (2002)	Elective cardiopulmonary bypass surgery	43 (33/10)	63.3 ± 2.3	N/A	Parallel	N/A	Enalapril	7.5-20 mg/day	7 days
Walters (2001)	Stroke with carotid disease	24 (15/9)	70.4 ± 8.4	N/A	Parallel	N/A	Perindopril	4 mg/day	2 weeks
Walters (2004)	Lacunar Infarction	12 (12/0)	63.2 ± 2.3	N/A	Cross-over	N/A	Perindopril	4 mg/day	2 weeks
Wang (2012)	Non-proliferative diabetic retinopathy	317 (179/138)	63.7 ± 8.7	N/A	Parallel	China	Captopril	25-37.5 mg/day	24 months
Wang (2016)	Atrial fibrillation recurrence	256 (160/96)	55.0 ± 35.0	N/A	Parallel	China	Perindopril	8 mg/day	1 years
Webster (1985a)	Chronic heart failure	20 (17/3)	N/A	N/A	Parallel	N/A	Enalapril	10 mg/day	12 weeks
Webster (1985b)	Chronic congestive heart failure	20 (17/3)	N/A	N/A	Parallel	N/A	Enalapril	10 mg/day	12 weeks
Weinberger (1983)	Hypertension	160 (101/79)	51.5 ± 5.0	N/A		United States	Captopril	25 and 75 mg/day	6 weeks
Weir and Saunders (1998)	Mild to moderate hypertension	322 (146/176)	53.1 ± 11.5	African-American	Parallel	N/A	Trandolapril	0.25, 0.5, 1, 2, 4, 8, 12, and 16 mg/day	6 weeks
West (1991)	Essential hypertension	13 (13/1)	53.4 ± 8.7	Caucasian	Cross-over	N/A	Benazapril	10 mg/day	6 weeks
White (1988)	Mild to moderate essential hypertension	18 (10/8)	52.0 ± 12.0	N/A	Parallel	United States	Cilazapril	2.5-5 mg/day	12 weeks
White (1995)	Ambulatory blood pressure monitoring	51 (43/8)	54.7 ± 4.3	White, Black, Hispanic, and Others	Parallel	N/A	Moexipril	7.5 and 15 mg/day	8 weeks
White (2002)	Hypertension	154 (88/57)	54.6 ± 10.3	Black and Non-black	Parallel	N/A	Enalapril	10-20 mg/day	8 weeks
Wiegmann (1992)	Type 1 diabetes	18 (N/A)	25.9 ± 9.7	N/A	Cross-over	United States	Captopril	50 mg/day	12 weeks

Wilkins (1983)	Mild low-renin essential hypertension	23 (2/21)	N/A	Black and White	Parallel	N/A	Enalapril	10-40 mg/day	12 weeks
Willenheimer (2001)	Stable ischemic heart disease	389 (13 8/251)	N/A	N/A	Parallel	N/A	Ramipril	1.25 and 5 mg/day	6 months
Willoughby (2012)	Ischemic heart disease or diabetes plus additional coronary risk factor	119 (73/46)	66.8 ± 10.1	N/A	Parallel	Australia	Ramipril	10 mg/day	12 weeks
Winer (1990)	None	8 (8/0)	N/A	N/A	N/A	United States	Enalapril	10 mg/day	5 days
Wyld (1994)	None	6 (6/0)	29.5 ± 11.0	N/A	Cross-over	United Kingdom	Idrapril	200, 400, and 800 mg/day	5 days
Yu (2006)	End-stage renal disease on regular hemodialysis therapy	46 (30/16)	46.4 ± 13.4	N/A	Parallel	Taiwan	Ramipril	3.75-7.5 mg/day	12 months
Yusuf (1991)	Congestive heart failure	2569 (2064/505)	N/A	Black, White, and Others	Parallel	United States, Canada, and Belgium	Enalapril	2.5-20 mg/day	41.4 months
Yusuf (1992)	Asymptomatic left ventricular dysfunction	4228 (3744/484)	N/A	Black, White, and Others	Parallel	United States, Canada, and Belgium	Enalapril	2.5-20 mg/day	37.4 months
Yusuf (2000)	Congestive heart failure	9297 (6817/2480)	66.0 ± 7.0	N/A	Factorial design	Canada, United States, western European, Argentina, Brazil, and Mexico	Ramipril	10 mg/day	5 years
Zamboulis (1996)	Mild to moderate essential hypertension	23 (15/8)	N/A	N/A	Parallel	N/A	Fosinopril	20 mg/day	4 weeks
Zannad (2006)	End-stage renal disease	397 (208/189)	67.0 ± 7.9	N/A	Parallel	France	Fosinopril	5-20 mg/day	24 months
Zee (1998)	A history of angiotensin-	169 (71/98)	57.8 ± 10.1	N/A	Cross-over	N/A	Lisinopril	20 mg/day	6 weeks

	converting enzyme inhibitors-associated cough								
Zeng (1998)	Neurally mediated syncope	30 (12/18)	37.6 ± 11.1	N/A	Parallel	China	Enalapril	10 mg/day	7 days
Zi (2003)	Diastolic heart failure	74 (26/48)	77.5 ± 9.9	N/A	Parallel	United Kingdom	Quinapril	5-40 mg/day	6 months

N/A, not available.
