# Supplementary material for: Adverse Effects of Angiotensin-Converting Enzyme Inhibitors in Humans: A Systematic Review and Meta-Analysis of 378 Randomized Controlled Trials
Source: Int J Environ Res Public Health. 2022 Jul 8;19(14):8373. doi: 10.3390/ijerph19148373 (PMC9324875; doi:10.3390/ijerph19148373)
Supplement: Supplementary file 1 [file ijerph-19-08373-s001.zip › Table S3.pdf]

Table S3 Subgroup analysis of dry cough outcome based on the drug name

Drug name	Number of studies	Intervention		Heterogeneity		Effect estimates		
		ACE inhibitor (events/total)	Placebo (events/total)	Chi	I^2 (%)	RR	95% CI	*p* value
Overall	99	4352/54518	2112/53220	<0.001	81.5	2.66	2.20 – 3.20	<0.001
Benazapril	3	4/528	3/510	0.586	0.0	1.24	0.28 – 5.53	0.776
Captopril	11	49/1678	14/1571	0.905	0.0	2.59	1.46 – 4.59	0.001
Cilazapril	3	6/94	0/63	0.969	0.0	3.70	0.66 – 20.90	0.138
Enalapril	25	1756/6402	1408/6219	0.580	0.0	1.23	1.16 – 1.30	<0.001
Fosinopril	4	21/333	14/325	0.586	0.0	1.32	0.68 – 2.55	0.418
Imidapril	1	2/15	0/15	-	-	-	-	-
Lisinopril	6	55/549	34/545	0.034	58.5	1.66	0.81 – 3.39	0.168
Moexipril	1	11/103	2/48	-	-	2.56	0.59 – 11.12	0.209
Perindopril	11	673/22761	152/22532	0.806	0.0	4.19	3.51 – 4.98	<0.001
Quinapril	8	340/2545	156/2416	0.141	35.9	2.71	1.63 – 4.51	<0.001
Ramipril	21	1404/18518	322/18391	0.321	10.6	4.13	3.58 – 4.78	<0.001
Spirapril	1	4/52	0/26	-	-	-	-	-
Temocapril	1	2/19	0/11	-	-	-	-	-
Trandolapril	5	25/921	7/616	0.994	0.0	2.45	1.11 – 5.42	0.026
