# Supplementary material for: Adverse Effects of Angiotensin-Converting Enzyme Inhibitors in Humans: A Systematic Review and Meta-Analysis of 378 Randomized Controlled Trials
Source: Int J Environ Res Public Health. 2022 Jul 8;19(14):8373. doi: 10.3390/ijerph19148373 (PMC9324875; doi:10.3390/ijerph19148373)
Supplement: Supplementary file 1 [file ijerph-19-08373-s001.zip › Table S4.pdf]

Table S4 Sensitivity analyses of low-risk-of-bias or some-concerned randomized-controlled trials of ACE inhibitors vs placebo on adverse outcomes

**Adverse outcomes**	**Number of studies**	**Intervention**		**Heterogeneity**		**Effect estimates**		
		**ACE inhibitors (events/total)**	**Placebo (events/total)**	**Chi**	**I^2 (%)**	**RR**	**95% CI**	***p* value**
Confirmatory approach								
Cough	95	3831/41418	1990/39993	<0.001	78.4	2.53	2.10-3.04	<0.001
Hypotension	39	693/16391	283/14694	0.180	17.2	2.08	1.71-2.52	<0.001
Hyperkalemia	17	139/6316	96/6125	0.523	0.0	1.24	1.01-1.51	0.041
Angioedema	11	134/15416	121/15441	0.310	14.1	1.16	0.84-1.60	0.359
Exploratory approach								
Cardiovascular problems								
Cardiac arrhythmia	8	99/3562	96/3311	0.498	0.0	0.99	0.75 – 1.29	0.915
Chest pain	28	288/8491	294/8335	0.571	0.0	0.95	0.82-1.11	0.540
Congestive heart failure	11	184/3767	209/3788	0.793	0.0	0.88	0.74 – 1.05	0.169
Flushing	5	21/444	11/425	0.017	66.6	3.18	0.49 – 20.48	0.223
Hypertension	13	285/15422	458/15414	0.308	13.6	0.64	0.53-0.77	<0.001
Myocardial infarction	20	66/6367	57/5466	0.915	0.0	0.8506	0.59-1.23	0.394
Palpitation or tachycardia	7	40/666	4/438	0.352	10.1	2.14	0.72 – 6.34	0.169
Reinfarction	4	15/472	15/473	0.271	23.4	0.87	0.27 – 2.81	0.820
Revascularization	5	258/4702	130/4006	0.061	55.7	0.82	0.55 – 1.25	0.358

Adverse outcomes	Number of studies	Intervention		Heterogeneity		Effect estimates		
		ACE inhibitors (events/total)	Placebo (events/total)	Chi	I^2 (%)	RR	95% CI	*p* value
Worsening heart failure	5	28/346	87/355	0.348	10.2	0.36	0.23 – 0.56	<0.001
Gastrointestinal problems								
Abdominal pain	10	37/507	7/334	0.480	0.0	1.65	0.74 – 3.67	0.220
Constipation	5	5/484	3/184	0.730	0.0	0.96	0.26 – 3.51	0.952
Diarrhea	9	70/3440	60/31	0.276	18.8	1.10	0.68 – 1.78	0.708
Flatulence	3	2/80	2/83	0.364	1.1	0.89	0.15 – 5.38	0.903
Gastroenteritis	4	4/301	6/182	0.316	15.3	0.61	0.16 – 2.33	0.467
Indigestion	3	5/233	2/148	0.106	55.4	1.29	0.09 – 17.68	0.847
Nausea & vomiting	21	93/2035	92/1363	0.916	0.0	0.78	0.59-1.02	0.067
Nonspecific gastrointestinal symptom	5	19/370	18/360	0.792	0.0	0.98	0.54 – 1.78	0.955
Integumentary problems								
Allergy	6	9/511	5/493	0.944	0.0	1.47	0.56 – 3.89	0.432
Cramp	4	3/369	2/158	0.298	18.5	0.91	0.17 – 4.87	0.910
Joint & muscle pain	6	33/457	10/255	0.254	24.0	1.87	0.79 – 4.45	0.154
Skin infection	4	23/267	26/256	0.248	27.3	1.04	0.32 – 3.37	0.947
Skin rash	22	48/2181	33/1664	0.976	0.0	1.15	0.75-1.76	0.528
Neuropsychiatry problems								

Adverse outcomes	Number of studies	Intervention		Heterogeneity		Effect estimates		
		ACE inhibitors (events/total)	Placebo (events/total)	Chi	I^2 (%)	RR	95% CI	*p* value
Anxiety	3	6/242	10/250	0.285	20.4	0.80	0.20 – 3.18	0.753
Dizziness	57	560/37776	264/35664	0.412	3.0	1.48	1.25-1.74	<0.001
Headache	65	851/38390	724/35625	0.272	9.1	0.95	0.82-1.10	0.478
Sleep disturbance	5	19/134	8/132	0.432	0.0	1.77	0.80 – 3.91	0.159
Stroke	6	7/1391	9/1282	0.772	0.0	0.73	0.27 – 1.93	0.522
Syncope	10	40/1630	21/1371	0.608	0.0	1.49	0.89 – 2.49	0.134
Respiratory problems								
Dyspnea	4	2/342	17/285	0.358	7.0	0.28	0.06 – 1.25	0.095
Respiratory infection	9	100/1366	58/949	0.306	15.3	1.27	0.89 – 1.81	0.194
Respiratory problems	10	46/1158	20/671	0.563	0.0	1.33	0.77 – 2.29	0.303
Renal problems & electrolyte imbalance								
Edema	11	67/4104	77/3978	0.348	10.1	0.81	0.54 – 1.19	0.281
Hypokalemia	5	7/309	24/307	0.106	47.5	0.47	0.11 – 2.00	0.307
Proteinuria	7	75/11824	88/11818	0.302	16.8	0.76	0.48 – 1.20	0.243
Renal impairment	19	432/43013	314/42851	<0.001	63.3	1.13	0.72 – 1.75	0.598
Urination	4	5/482	3/270	0.162	41.61	0.92	0.14 – 6.11	0.933
Miscellaneous								
Cancer	11	76/4833	68/4707	0.468	0.0	1.09	0.79 – 1.52	0.588

Adverse outcomes	Number of studies	Intervention		Heterogeneity		Effect estimates		
		ACE inhibitors (events/total)	Placebo (events/total)	Chi	I^2 (%)	RR	95% CI	*p* value
Erectile dysfunction or impotence	4	3/776	5/322	0.485	0.0	0.36	0.09 – 1.45	0.151
Fatigue	35	188/3459	102/2233	0.40	4.0	1.18	0.92-1.50	0.185
Flu symptoms	3	11/119	3/93	0.165	44.5	0.81	0.08 – 8.16	0.861
Inflammation	5	24/250	13/219	0.816	0.0	1.68	0.94 – 3.01	0.082
Malaise	4	9/149	4/118	0.780	0.0	1.64	0.54 – 4.92	0.381
Neutropenia	4	17/761	21/754	0.834	0.0	0.80	0.44 – 1.46	0.464
Stress	3	8/127	5/116	0.539	0.0	1.31	0.43 – 3.97	0.629
Sweating	3	1/223	4/110	0.428	0.0	0.31	0.06 – 1.68	0.172
