# Supplementary figures and images for: Adverse Effects of Angiotensin-Converting Enzyme Inhibitors in Humans: A Systematic Review and Meta-Analysis of 378 Randomized Controlled Trials
Source: Int J Environ Res Public Health. 2022 Jul 8;19(14):8373. doi: 10.3390/ijerph19148373 (PMC9324875; doi:10.3390/ijerph19148373)

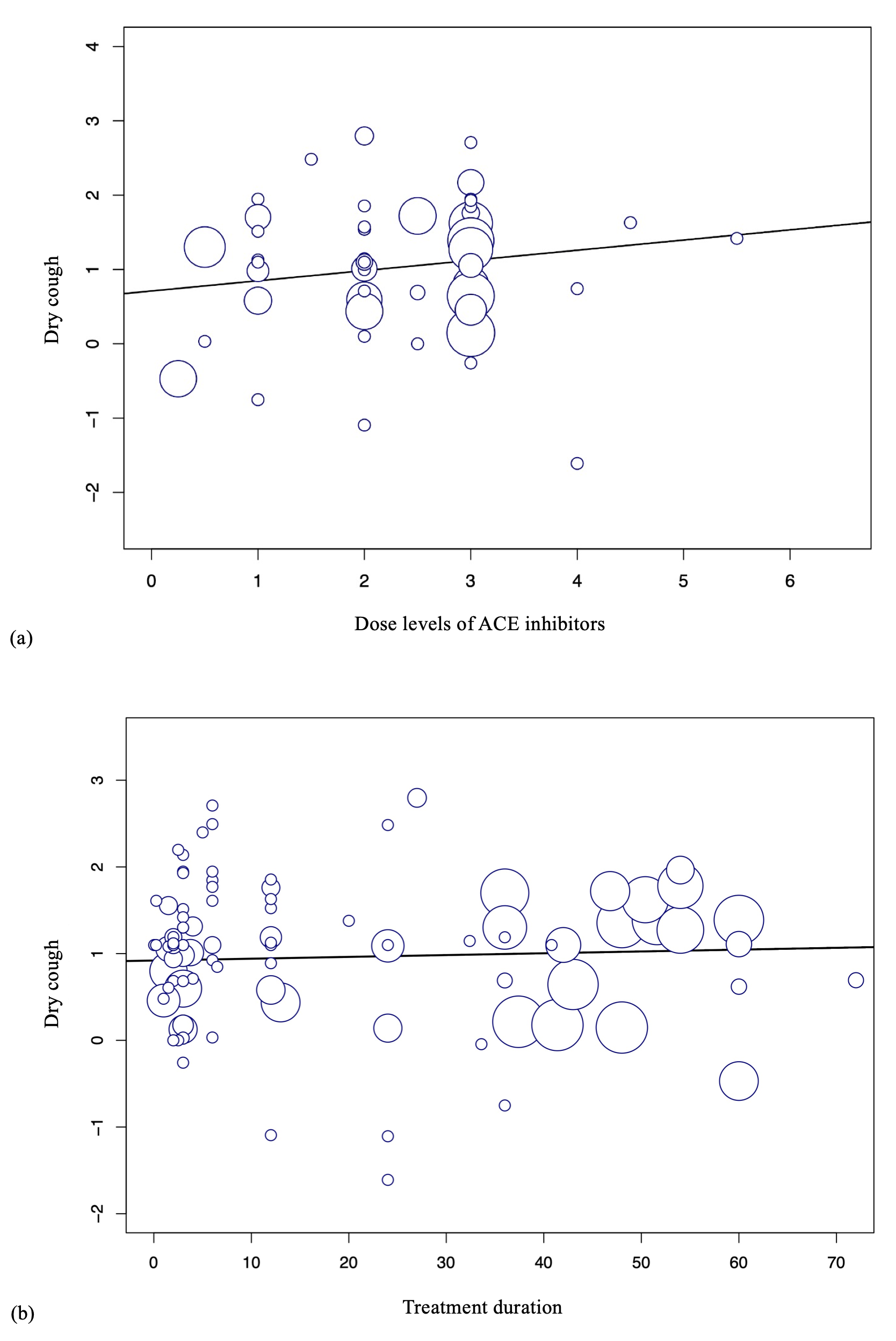

Supplement: Supplementary file 1 [file ijerph-19-08373-s001.zip › Figure S2.tiff]
